# Association Between Atopic Dermatitis and Educational Attainment in Denmark

**DOI:** 10.1001/jamadermatol.2021.0009

**Published:** 2021-04-14

**Authors:** Sigrun Alba Johannesdottir Schmidt, Aurélie Mailhac, Bianka Darvalics, Amy Mulick, Mette S. Deleuran, Henrik T. Sørensen, Jette Lindorff Riis, Sinéad M. Langan

**Affiliations:** 1 Department of Clinical Epidemiology, Aarhus University Hospital, Aarhus, Denmark; 2 Department of Dermatology, Aarhus University Hospital, Aarhus, Denmark; 3 Faculty of Epidemiology and Population Health, London School of Hygiene and Tropical Medicine, London, United Kingdom; 4 Health Data Research UK, London, United Kingdom

## Abstract

**Importance:**

Atopic dermatitis (AD) may affect academic performance through multiple pathways, including poor concentration associated with itching, sleep deprivation, or adverse effects of medications. Because educational attainment is associated with health and well-being, any association with a prevalent condition such as AD is of major importance.

**Objective:**

To examine whether a childhood diagnosis of AD is associated with lower educational attainment.

**Design, Setting, and Participants:**

This population-based cohort study used linked routine health care data from January 1, 1977, to June 30, 2017 (end of registry follow-up), in Denmark. The study population included all children born in Denmark on June 30, 1987, or earlier with an inpatient or outpatient hospital clinic diagnosis of AD recorded before their 13th birthday (baseline) and a comparison cohort of children from the general population matched by birth year and sex. A secondary analysis included exposure-discordant full siblings as a comparison cohort to account for familial factors. Data were analyzed from September 11, 2019, to January 21, 2021.

**Exposures:**

Hospital-diagnosed AD.

**Main Outcomes and Measures:**

Estimated probability or risk of not attaining specific educational levels (lower secondary, upper secondary, and higher) by 30 years of age among children with AD compared with children in the matched general population cohort. Corresponding risk ratios (RRs) were computed using Poisson regression that was conditioned on matched sets and adjusted for age. The sibling analysis was conditioned on family and adjusted for sex and age.

**Results:**

The study included a total of 61 153 children, 5927 in the AD cohort (3341 male [56.4%]) and 55 226 from the general population (31 182 male [56.5%]). Compared with matched children from the general population, children with AD were at increased risk of not attaining lower secondary education (150 of 5927 [2.5%] vs 924 of 55 226 [1.7%]; adjusted RR, 1.50; 95% CI, 1.26-1.78) and upper secondary education (1141 of 5777 [19.8%] vs 8690 of 52 899 [16.4%]; RR, 1.16; 95% CI, 1.09-1.24), but not higher education (2406 of 4636 [51.9%] vs 18 785 of 35 408 [53.1%]; RR, 0.95; 95% CI, 0.91-1.00). The absolute differences in probability were less than 3.5%. The comparison of 3259 children with AD and 4046 of their full siblings yielded estimates that were less pronounced than those in the main analysis (adjusted RR for lower secondary education, 1.29 [95% CI, 0.92-1.82]; adjusted RR for upper secondary education, 1.05 [95% CI, 0.93-1.18]; adjusted RR for higher education, 0.94 [95% CI, 0.87-1.02]).

**Conclusions and Relevance:**

This population-based cohort study found that hospital-diagnosed AD was associated with reduced educational attainment, but the clinical importance was uncertain owing to small absolute differences and possible confounding by familial factors in this study. Future studies should examine for replicability in other populations and variation by AD phenotype.

## Key Points

**Question:**

Is atopic dermatitis (AD) associated with lower educational attainment?

**Findings:**

This nationwide matched cohort study of 61 153 Danish children found evidence of reduced attainment of lower and upper secondary education in 5927 children with a hospital diagnosis of AD compared with 55 226 matched children from the general population; however, absolute differences were less than 3.5%. Estimates were less pronounced in a secondary analysis comparing patients with their full siblings.

**Meaning:**

Hospital-diagnosed AD is associated with reduced educational attainment, but the clinical importance is uncertain owing to small absolute differences and possible confounding by familial factors.

## Teaser

This population-based cohort study examined whether a childhood diagnosis of atopic dermatitis is associated with lower educational attainment among children in Denmark.

## Introduction

Atopic dermatitis (AD) affects 20% of children in Western countries and is characterized by recurrent pruritic eczematous lesions.^1^ Atopic dermatitis may affect academic performance through several pathways. Itching, sleep deprivation, or adverse effects of sedating antihistamines may cause school absenteeism and “presenteeism” (ie, being present at school, but without fully functioning).^1,2,3^ Atopic comorbidity could add to these effects, as may psychological distress due to AD stigma.^1,2,4^ Increased risk of depression, attention-deficit/hyperactivity disorder, and learning disabilities may also mediate reduced academic performance in children with AD.^1,2,4,5,6,7,8^

Because educational attainment is associated with health and well-being,^9^ any association of educational attainment with AD is important. Nevertheless, the evidence is limited.^10,11^ A Dutch cohort study^10^ found no association between AD in childhood and standardized indicators of appropriate secondary school level at 11 years of age. In a Swedish cohort study of male military conscripts,^11^ adult AD was associated with a higher chance of attaining university but not high school education compared with 9 years of compulsory lower secondary education. Small sample sizes,^10,11^ short follow-up,^10^ missing data,^10^ restriction to male participants,^11^ and lack of absolute effect measures preclude firm study conclusions. A particular concern is confounding by familial factors (eg, parental socioeconomic status, family structure, and genetic and environmental factors),^10,12^ which can be difficult to eliminate.

We used routinely collected health data from Denmark to conduct a cohort study on whether children with hospital-diagnosed AD had lower educational attainment than children from the general population. In a secondary analysis, we used patients’ full siblings as a comparison group to control for familial factors. We assessed age at AD diagnosis, sex, and family socioeconomic status as potential effect modifiers.

## Methods

### Setting

This population-based cohort study was approved by the Danish Data Protection Agency. Danish legislation does not require approval by an ethical review board or informed consent from patients for registry-based studies. This study followed the Reporting of Studies Conducted Using Observational Routinely Collected Health Data (RECORD) reporting guideline. The study protocol is available in Supplement 1.

The Danish welfare model promotes health and social equity through universal access to health care and education.^13,14^ We used nationwide data from the Danish National Patient Registry^15^ and Psychiatric Central Research Registry (diagnoses from hospital admissions or appointments),^16^ the Danish Civil Registration System (demographics, death, migration, and close kinship),^17^ the Danish National Prescription Registry (filled prescriptions),^18^ the Danish Medical Birth Registry (birth outcomes),^19^ and Statistics Denmark (educational level and income).^20,21^ We linked data using unique personal identifiers.^17^ We provide a detailed description of the setting and variable definitions in eMethods 1 in Supplement 2.

### Study Cohorts

Using the Danish National Patient Registry, we identified children who received a primary or secondary hospital (inpatient, outpatient, or emergency department) diagnosis of AD between January 1, 1977, and June 30, 2000. The date of the first admission or hospital outpatient (ambulatory) appointment was considered the diagnosis date. We restricted the analysis to children who were diagnosed before 13 years of age and born June 30, 1987, or earlier, which ensured a minimum age of 30 years on the last date of registry follow-up (June 30, 2017). We further restricted the analysis to children who were born in Denmark and were living in Denmark at the time of diagnosis to ensure availability of registry data.

For the main analysis, we used the Civil Registration System to identify a matched general population comparison cohort.^22^ Thus, for each child with AD, we randomly sampled up to 10 children among persons who had the same sex, were born in Denmark in the same year, and who were alive and living in Denmark with no recorded AD diagnosis on the diagnosis date for the exposed child (ie, risk-set sampling).

In a secondary (sibling) analysis, exposure-discordant full siblings (ie, full siblings without a record of AD) served as comparators, which controlled for stable confounders and mediators shared in a family.^23^ This analysis thus estimates the direct study effect. We assumed that there was a stronger correlation between familial factors than AD diagnoses among siblings^23^ and that exposure or outcome for one sibling did not affect that of other siblings (ie, absence of sibling carryover).^24^

### Outcomes

**Figure 1.**
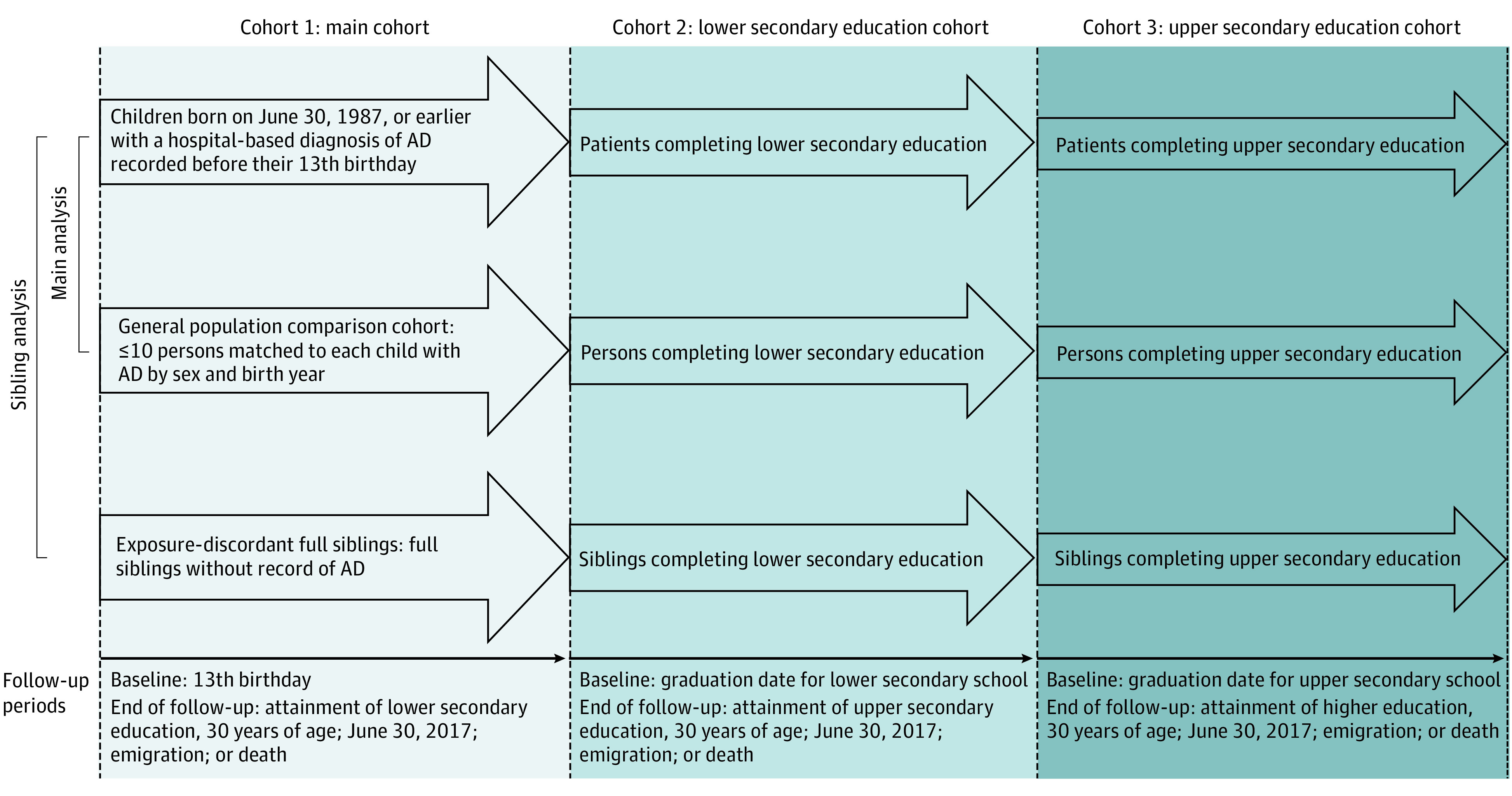
Illustration of Study Design, Including Definitions of Cohorts and Follow-up Periods AD indicates atopic dermatitis.

We identified the highest level of educational attainment by 30 years of age recorded in the Population Education Registry^20^ using the Danish nomenclature of education: lower secondary education (International Standard Classification of Education levels 1-2), upper secondary education (International Standard Classification of Education level 3), and higher (ie, tertiary/university) education (International Standard Classification of Education levels 5-8).^14,25,26^ For each educational level, we generated a binary outcome variable coded as 1 for those who did not attain that level and 0 for those who did. Each level was conditional on completing the preceding levels. To account for this conditionality and time-related changes in covariables, we defined 3 cohorts for assessing attainment of each educational level (Figure 1):

1. Main cohort (outcome, lower secondary education): AD and comparison cohorts described above. Baseline was the 13th birthday.
2. Lower secondary education cohort (outcome, upper secondary education): members of the main cohort who completed lower secondary education by 30 years of age. Baseline was graduation date for lower secondary school.
3. Upper secondary education cohort (outcome, higher education): further restricted to those who completed upper secondary education by 30 years of age. Baseline was graduation date for upper secondary school.

### Statistical Analysis

Data were analyzed from September 11, 2019, to January 21, 2021. We produced descriptive statistics at baseline for all cohorts. We computed the probability or risk of not attaining each educational level outcome by 30 years of age. We used conditional Poisson regression (as recommended by Cummings^27^) to estimate the corresponding risk ratio (RR) with 95% CIs among children with AD compared with the matched general population cohort and with full siblings. In the main analysis, the unadjusted model accounted for sex and birth year by conditioning on matched sets. We adjusted additionally for age at baseline (age was 13 years for all persons in the main cohort). In the sibling analysis, we conditioned on family in the unadjusted model to allow within-family comparisons and then additionally adjusted for sex and age at baseline. A stratum could include more than 1 child with AD and all exposure-discordant full siblings. To illustrate whether patients with AD required more time to attain a given educational level, we plotted the probability of attaining specific educational levels by age. As a secondary aim, we examined the probability of attaining subtypes of upper secondary education (general or vocational) and higher education (short, medium, or long cycle).

We stratified the results by age at diagnosis (0-4 and 5-12 years), sex, and, for the sibling analysis, parental income and educational level. We conducted several sensitivity analyses detailed in eMethods 2 in Supplement 2. We performed analyses using SAS, version 9.4 (SAS Institute, Inc).

## Results

The study included 61 153 children, 5927 with AD (3341 male [56.4%] and 2586 female [43.6%]) vs 55 226 general population comparators (31 182 male [56.5%] and 24 044 female [43.5%]) in the main cohorts, 5777 vs 52 899 in the lower secondary education cohorts, and 4636 vs 35 408 in the upper secondary education cohorts (flowchart provided in eFigure 1 in Supplement 2). In the sibling analysis, the number of children with AD and full siblings was 3259 and 4046, respectively, in the main cohorts, 3144 and 3877, respectively, in the lower secondary education cohort, and 2309 and 2746, respectively, in the upper secondary education cohort (eFigure 2 in Supplement 2). Two-thirds of children with AD were diagnosed before 5 years of age (4002 [67.5%]) (Table). Most were diagnosed before the 1990s (5113 [86.3%]) and at admission (5329 [89.9%]). Asthma (2162 [36.5%] vs 1560 [2.8%]) and rhinitis (540 [9.1%] vs 188 [0.3%]) were more prevalent among patients with AD than among general population comparators; other covariables were equally distributed. The sibling analysis yielded similar descriptive data, except that the AD cohorts included a higher proportion of male participants (3341 [56.4%]) than the sibling cohorts (1870 [57.4%]) and that siblings had a higher prevalence of asthma (221 [5.5%]) and rhinitis (38 [0.9%]) than the general population comparators (Table).

**Table.** Baseline Characteristics of Children With AD, a Matched General Population Comparison Cohort, and Full Siblings Without AD

Variable	No. (%) of children						
	Main cohort			Secondary education cohort			
				Lower		Upper	
	AD cohort	Comparison cohort	AD cohort		Comparison cohort	AD cohort	Comparison cohort
**Main analysis**							
Total	5927 (100)	55 226 (100)	5777 (100)		52 899 (100)	4636 (100)	35 408 (100)
Birth year							
1973-1977	1699 (28.7)	16 100 (29.2)	1660 (28.7)		15 483 (29.3)	1336 (28.8)	10 360 (29.3)
1978-1982	1923 (32.4)	18 291 (33.1)	1881 (32.6)		17 589 (33.3)	1479 (31.9)	11 581 (32.7)
1983-1987	2305 (38.9)	20 835 (37.7)	2236 (38.7)		19 827 (37.5)	1821 (39.3)	13 467 (38.0)
Age at AD diagnosis, y							
0-4	4002 (67.5)	NA	3888 (67.3)		NA	3041 (65.6)	NA
5-12	1925 (32.5)	NA	1889 (32.7)		NA	1595 (34.4)	NA
Sex							
Male	3341 (56.4)	31 182 (56.5)	3241 (56.1)		29 552 (55.8)	2521 (54.4)	18 638 (52.6)
Female	2586 (43.6)	24 044 (43.5)	2536 (43.9)		23 347 (44.1)	2115 (45.6)	16 770 (47.4)
Calendar period of AD diagnosis							
1976-1980	1445 (24.4)	NA	1405 (24.3)		NA	1075 (23.2)	NA
1981-1985	2010 (33.9)	NA	1958 (33.9)		NA	1566 (33.8)	NA
1986-1990	1658 (28.0)	NA	1617 (28.0)		NA	1321 (28.5)	NA
1991-1995	628 (10.6)	NA	<5^a^		NA	517 (11.2)	NA
1996-2000	186 (3.1)	NA	<5^a^		NA	157 (3.4)	NA
Setting of AD diagnosis							
Inpatient	5329 (89.9)	NA	5193 (89.9)		NA	4142 (89.3)	NA
Outpatient clinic	583 (9.8)	NA	569 (9.8)		NA	484 (10.4)	NA
Emergency department	15 (0.3)	NA	15 (0.3)		NA	10 (0.2)	NA
ADHD, depression, or anxiety disorder	16 (0.3)	65 (0.1)	22 (0.4)		134 (0.3)	48 (1.0)	309 (0.9)
Epilepsy	105 (1.8)	525 (1.0)	112 (1.9)		592 (1.1)	72 (1.6)	368 (1.0)
Asthma	2162 (36.5)	1560 (2.8)	2437 (42.2)		2486 (4.7)	2304 (49.7)	2852 (8.1)
Rhinitis	540 (9.1)	188 (0.3)	655 (11.3)		280 (0.5)	682 (14.7)	294 (0.8)
5-min Apgar score <7 or intrauterine/birth asphyxia	308 (5.2)	2249 (4.1)	296 (5.1)		2151 (4.1)	219 (4.7)	1368 (3.9)
Preterm birth (gestational age <37 wk)	215 (3.6)	1667 (3.0)	204 (3.5)		1591 (3.0)	143 (3.1)	998 (2.8)
Low birth weight (<2500 g)	347 (5.9)	2927 (5.3)	333 (5.8)		2812 (5.3)	216 (4.7)	1747 (4.9)
**Sibling analysis**							
Total	3259 (100)	4046 (100)	3144 (100)		3877 (100)	2309 (100)	2746 (100)
Birth year							
1973-1977	862 (26.4)	1265 (31.3)	832 (26.5)		1217 (31.4)	622 (26.9)	863 (31.4)
1978-1982	1255 (38.5)	1660 (41.0)	1213 (38.6)		1593 (41.1)	881 (38.2)	1121 (40.8)
1983-1987	1142 (35.0)	1121 (27.7)	1099 (35.0)		1067 (27.5)	806 (34.9)	762 (27.7)
Age at AD diagnosis, y							
0-4	2213 (67.9)	NA	2123 (67.5)		NA	1525 (66.0)	NA
5-12	1046 (32.1)	NA	1021 (32.5)		NA	784 (34.0)	NA
Sex							
Male	1870 (57.4)	2071 (51.2)	1798 (57.2)		1974 (50.9)	1277 (55.3)	1365 (49.7)
Female	1389 (42.6)	1975 (48.8)	1346 (42.8)		1903 (49.1)	1032 (44.7)	1381 (50.3)
Calendar period of AD diagnosis							
1976-1980	771 (23.7)	NA	738 (23.5)		NA	528 (22.9)	NA
1981-1985	1198 (36.8)	NA	1156 (36.8)		NA	843 (36.5)	NA
1986-1990	882 (27.1)	NA	852 (27.1)		NA	639 (27.7)	NA
1991-1995	321 (9.8)	NA	<5^a^		NA	233 (10.1)	NA
1996-2000	87 (2.7)	NA	<5^a^		NA	66 (2.9)	NA
Setting of AD diagnosis							
Inpatient	2961 (90.9)	NA	2856 (90.8)		NA	2092 (90.6)	NA
Outpatient clinic	292 (9.0)	NA	282 (9.0)		NA	<5^a^	NA
Emergency department	6 (0.2)	NA	6 (0.2)		NA	<5^a^	NA
ADHD, depression, or anxiety disorder	6 (0.2)	6 (0.1)	<5^a^		<5^a^	17 (0.7)	17 (0.6)
Epilepsy	52 (1.6)	50 (1.2)	<5^a^		<5^a^	26 (1.1)	30 (1.1)
Asthma	1153 (35.4)	221 (5.5)	1308 (41.6)		317 (8.2)	1158 (50.2)	362 (13.2)
Rhinitis	295 (9.1)	38 (0.9)	345 (11.0)		54 (1.4)	329 (14.2)	61 (2.2)
5-min Apgar score <7 or intrauterine/birth asphyxia	133 (4.1)	163 (4.0)	126 (4.0)		149 (3.8)	81 (3.5)	102 (3.7)
Preterm birth (gestational age <37 wk)	115 (3.5)	121 (3.0)	108 (3.4)		112 (2.9)	66 (2.9)	68 (2.5)
Low birth weight (<2500 g)	175 (5.4)	215 (5.3)	167 (5.3)		198 (5.1)	92 (4.0)	122 (4.4)

Abbreviations: AD, atopic dermatitis; ADHD, attention-deficit/hyperactivity disorder.

^a^ Not shown to preserve confidentiality.

**Figure 2.**
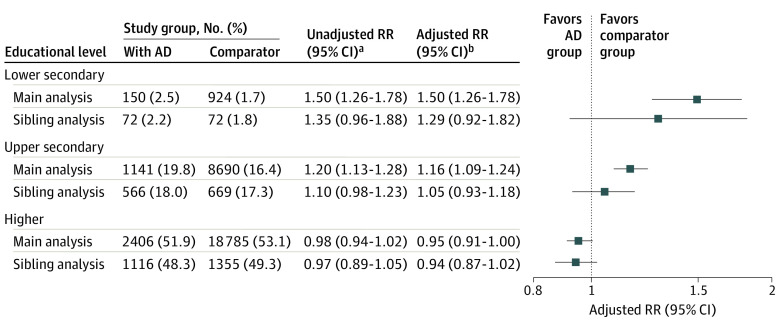
Probabilities and Risk Ratios (RRs) for Not Attaining Specific Educational Levels Children with atopic dermatitis (AD) are compared with a matched general population comparison cohort (main analysis) and full siblings without AD (sibling analysis). ^a^Accounting for sex in the main analysis and family in the sibling analysis. Age at baseline was the same for all members of the main cohort. ^b^Additionally adjusted for age at baseline and, in the sibling analysis, for sex.

**Figure 3.**
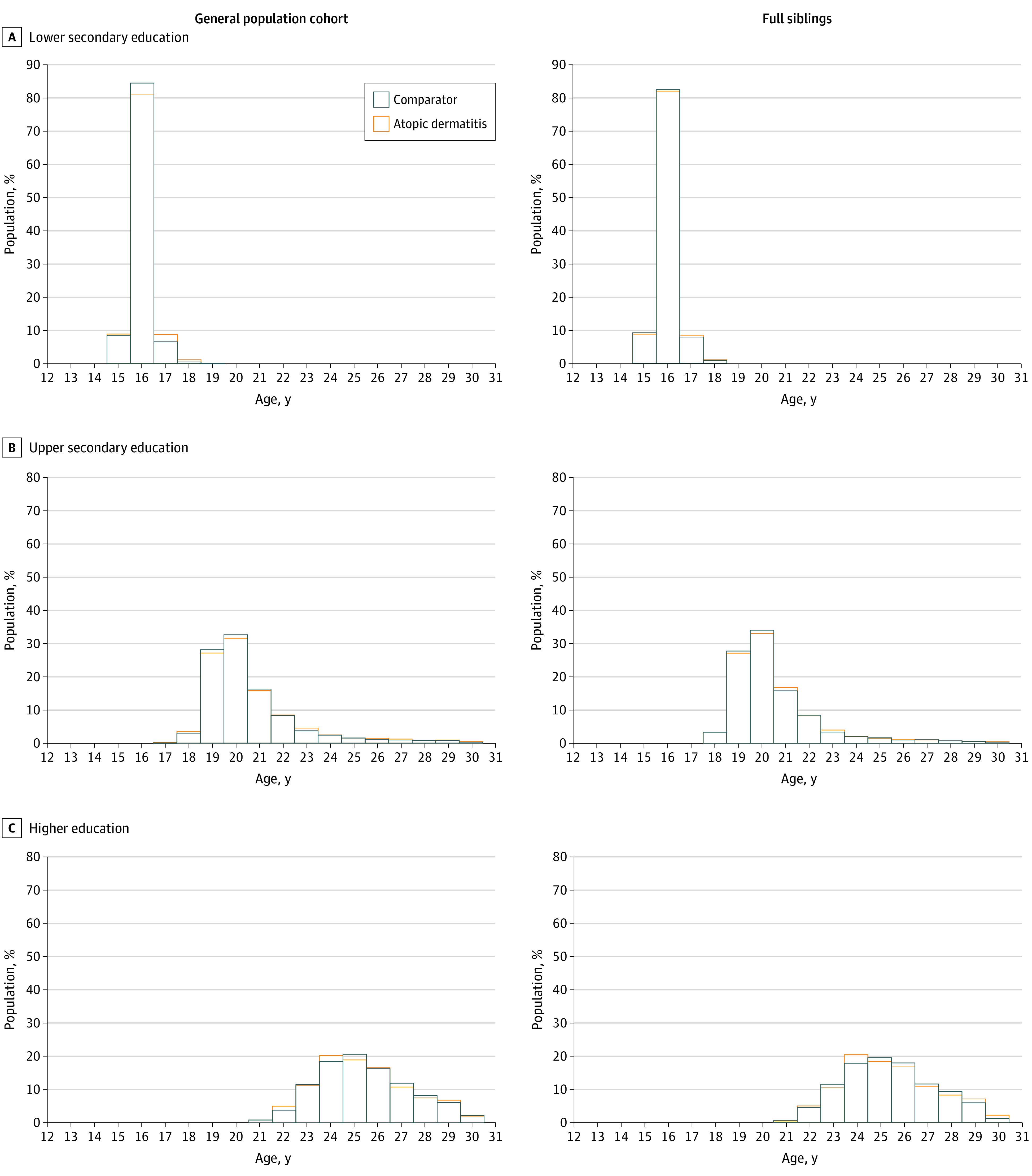
Histogram Showing the Probability of Attaining a Specific Educational Level Children with atopic dermatitis (AD) are compared with a matched general population cohort (left side) and full siblings without AD (right side) and stratified by age.

Children with AD had increased risk of not attaining lower secondary education (150 of 5927 [2.5%] vs 924 of 55 226 [1.7%]; adjusted RR, 1.50; 95% CI, 1.26-1.78) or upper secondary education (1141 of 5777 [19.8%] vs 8690 of 52 899 [16.4%]; adjusted RR, 1.16; 95% CI, 1.09-1.24) compared with general population comparators (Figure 2). This was not the case for higher education (2406 of 4636 [51.9%] vs 18 785 of 35 408 [53.1%]; adjusted RR, 0.95; 95% CI, 0.91-1.00). The absolute difference in probability of educational attainment for patients with AD vs comparators was less than 3.5% for all outcomes. Probabilities of attaining specific educational levels by a given age were similar for cohorts (Figure 3). There was also no substantial difference in probability of attaining subtypes of upper secondary education (2948/5777 [51.0%] vs 28 038/52 899 [53.0%] for general upper secondary education and 1688/5777 [29.2%] vs 16 171/52 899 [30.6%] for vocational upper secondary education in children with AD and comparators, respectively) and higher education (384/4636 [8.3%] vs 3049/35 408 [8.6%] for short-cycle, 875/4636 [18.9%] vs 6584/35 408 [18.6%] for medium-cycle, and 971/4636 [20.9%] vs 6990/35 408 [19.7%] for long-cycle higher education in patients with AD and comparators, respectively) by 30 years of age (eTable 1 in Supplement 2). The sibling analysis revealed associations that were less pronounced than those from the main analysis (adjusted RR for lower secondary education, 1.29 [95% CI, 0.92-1.82]; adjusted RR for upper secondary education, 1.05 [95% CI, 0.93-1.18]; adjusted RR for higher education, 0.94 [95% CI, 0.87-1.02]).

**Figure 4.**
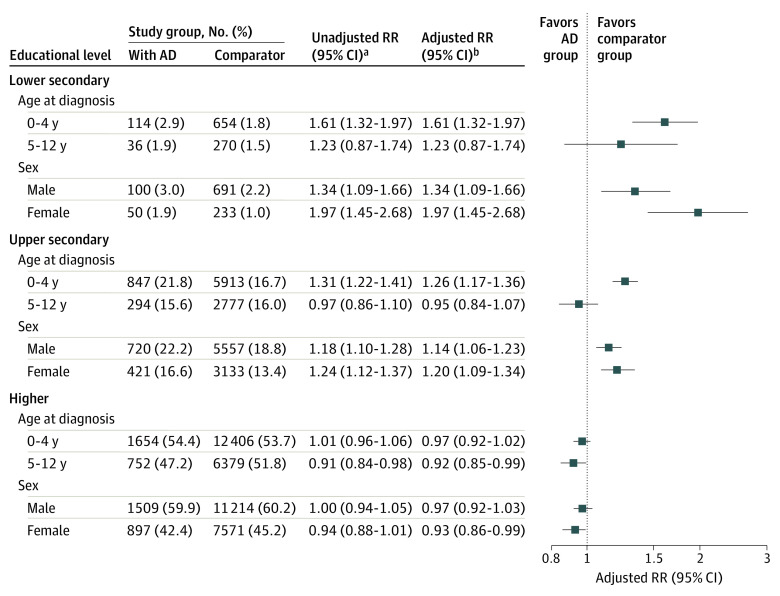
Stratified Probabilities and Risk Ratios (RRs) for Not Attaining Specific Educational Levels by Age and Sex Children with atopic dermatitis (AD) are compared with a matched general population cohort, stratified by age at AD diagnosis and sex. ^a^Accounting for sex in the main analysis. Age at baseline is the same for all members of the main cohort. ^b^Additionally adjusted for age at baseline.

The increased RR of not attaining lower secondary education was more pronounced for children diagnosed with AD before 5 years of age (adjusted RR, 1.61 [95% CI, 1.32-1.97] vs 1.23 [95% CI, 0.87-1.74]) and girls (adjusted RR, 1.97 [95% CI, 1.45-2.68] vs 1.34 [95% CI, 1.09-1.66]), but the absolute difference in probability between patients with AD and comparators was less than 1.2% for all subgroups (Figure 4). The increased risk of not attaining upper secondary education in children with AD compared with general population comparators was limited to children diagnosed before 5 years of age (probability, 847 of 3888 [21.8%] vs 5913 of 35 524 [16.7%]; RR, 1.26; 95% CI, 1.17-1.36). The sibling analysis yielded no effect measure modification by age, sex, or parental socioeconomic status (eFigure 3 in Supplement 2).

Findings of sensitivity analyses are shown in eTable 2 in Supplement 2. Results were robust for the main comparison, except that estimates decreased slightly when excluding patients with AD (and their general population comparators) who did not contribute to the sibling analysis (adjusted RR for lower secondary education, 1.34 [95% CI, 1.05-1.72]; adjusted RR for upper secondary education, 1.09 [95% CI, 1.00-1.19]; adjusted RR for higher education, 0.94 [95% CI, 0.89-1.00]). For the sibling comparison, the association with lower secondary education became slightly more pronounced in all sensitivity analyses (highest RR observed when assessing outcome at 35 years of age, 1.58 [95% CI, 0.98–2.56]), but the absolute reductions in probability remained minor. Strong correlations were found for both parental income (80.5%) and educational level (99.1%) between exposure-discordant siblings (eTable 3 in Supplement 2).

## Discussion

We found a higher risk of not attaining lower and upper secondary education in children with hospital-diagnosed AD compared with matched children from the general population. However, absolute reductions in probability were small and associations were less pronounced when compared with full siblings, suggesting confounding by familial factors.

In a Dutch cohort study including 1865 children,^10^ AD (which was assessed at several time points throughout childhood) was not associated with measures of school performance (Central Institute for Test Development tests and teachers’ school-level assessments) at 11 years of age, after adjusting for parental educational attainment, various health indicators, and sex. One or more repeated measurements were missing in 78%, and there was no follow-up beyond 11 years of age. In a Swedish study of 234 715 men who underwent compulsory military conscription between 1969 and 1976,^11^ AD was associated with higher cognitive function at conscription (β coefficient, 0.15; 95% CI, 0.05-0.24) and chance of completing university education (RR, 1.29; 95% CI, 1.13-1.47; reference lower secondary education); however, associations attenuated after adjusting for familial socioeconomic characteristics (β coefficient, 0.06 [95% CI, 0.03-0.15] and RR, 1.16 [95% CI, 1.00-1.35], respectively). There was no association with attaining upper secondary education (fully adjusted RR, 1.03; 95% CI, 0.91-1.18). Absolute risk measures were not reported. The study likely captured predominantly severe and persistent AD, because prevalence was only 0.7% and visible eczema was required on physical examination.

Our study supports the findings of previous studies,^10,11^ and the unique sibling design addresses concerns about confounding by familial factors. The attenuation of RRs when restricting the main comparison to strata where the patients with AD also contributed to the sibling analysis suggests possible confounding by family structure. Although the increased risk of not attaining upper secondary education was more pronounced in children diagnosed with AD before 5 years of age (a negative prognostic factor), consistent results were not obtained for lower secondary and higher education or in the sibling analysis. We also found no evidence of variation by sex or family socioeconomic status. However, we observed lower statistical precision in the sibling analysis, especially for subgroups.

### Strengths and Limitations

Our population-based cohort study design within a uniform welfare system with virtually complete long-term follow-up reduces selection bias.^13^ Selection based on socioeconomic factors that could determine need for hospital care for AD (eg, through poor management) and reduced educational attainment may occur but is controlled for in the sibling analysis.

The validity of AD diagnoses is likely high (positive predictive value of 99% from specialist departments^28^). However, incompleteness is expected because diagnoses from general practitioners and private practice dermatologists were unavailable. The comparison cohorts therefore may have included (mainly milder) AD cases, causing underestimates.

Improvement in AD with age could explain the decrease in RR with increasing educational level. Another explanation is self-selection of patients with AD—that is, persons who persevere in lower secondary education despite severe AD may be more determined to pursue higher education. Nevertheless, the absolute difference was minor even for lower secondary education, where time-related improvements in severity or selection bias would be least pronounced.

Education data are considered complete and valid.^20^ Although missing data from studies abroad is possible, emigration was similar in cohorts.

Residual confounding from genetic traits may occur in the sibling analysis, because the Civil Registration System records children under their legal parents.^17^ A carryover effect between siblings may have caused underestimates if academic performance of siblings is affected through family distress.^1,29,30^ The assumption that familial factors are more commonly shared by siblings is unverifiable, but within-family correlations for certain measured factors were strong in our sensitivity analysis.

The outcomes in this hospital-based cohort seem reassuring for patients with milder cases and in more recent calendar periods, because effective treatments for severe AD have emerged^1^ and the educational level has increased for the Danish population in general.^31^

## Conclusions

We found no clinically important difference in educational attainment in Danish children with hospital-diagnosed AD compared with children from the general population and full siblings without a medical record of AD, except for a small absolute reduction in attainment of lower secondary education. Future studies should examine for replicability in children with milder AD or in countries with less government support for health care and education. Variation by AD phenotype and comorbidities (eg, hand eczema) and associations with other measures of academic success (eg, grades) also require investigation.

## Notes

Supplement 1. Trial Protocol.

Supplement 2. **eMethods 1.** Description of Study Setting and Definitions of Variables **eMethods 2.** Details of Sensitivity Analyses **eFigure 1.** Flowchart for Main Analysis **eFigure 2.** Flowchart for Sibling Analysis **eFigure 3.** Probabilities and Risk Ratios (RRs) for not Attaining Specific Educational Levels **eTable 1.** Probability of Subtypes of Upper Secondary Education and Higher Education **eTable 2.** Probability and Risk Ratios (RRs) for not Attaining Specific Educational Levels **eTable 3.** Correlation Between Parental Income and Parental Educational Level Among Exposure-Discordant Siblings eReferences.

## References

[1] Weidinger S, Beck LA, Bieber T, Kabashima K, Irvine AD. Atopic dermatitis. Nat Rev Dis Primers. 2018;4(1):1-20. doi:10.1038/s41572-018-0001-z 29930242

[2] Silverberg JI. Selected comorbidities of atopic dermatitis: atopy, neuropsychiatric, and musculoskeletal disorders. Clin Dermatol. 2017;35(4):360-366. doi:10.1016/j.clindermatol.2017.03.008 28709566 PMC5512438

[3] Gruber R, Somerville G, Enros P, Paquin S, Kestler M, Gillies-Poitras E. Sleep efficiency (but not sleep duration) of healthy school-age children is associated with grades in math and languages. Sleep Med. 2014;15(12):1517-1525. doi:10.1016/j.sleep.2014.08.009 25441747

[4] Miyazaki C, Koyama M, Ota E, . Allergic diseases in children with attention deficit hyperactivity disorder: a systematic review and meta-analysis. BMC Psychiatry. 2017;17(1):120. doi:10.1186/s12888-017-1281-7 28359274 PMC5374627

[5] Patel KR, Immaneni S, Singam V, Rastogi S, Silverberg JI. Association between atopic dermatitis, depression, and suicidal ideation: a systematic review and meta-analysis. J Am Acad Dermatol. 2019;80(2):402-410. doi:10.1016/j.jaad.2018.08.063 30365995

[6] Young S, Hollingdale J, Absoud M, . Guidance for identification and treatment of individuals with attention deficit/hyperactivity disorder and autism spectrum disorder based upon expert consensus. BMC Med. 2020;18(1):146. doi:10.1186/s12916-020-01585-y 32448170 PMC7247165

[7] Fleming M, Fitton CA, Steiner MFC, . Educational and health outcomes of children and adolescents receiving antidepressant medication: Scotland-wide retrospective record linkage cohort study of 766 237 schoolchildren. Int J Epidemiol. 2020;49(4):1380-1391. doi:10.1093/ije/dyaa002 32073627 PMC7660154

[8] Wan J, Shin DB, Gelfand JM. Association between atopic dermatitis and learning disability in children. J Allergy Clin Immunol Pract. 2020;8(8):2808-2810. doi:10.1016/j.jaip.2020.04.032 32348912 PMC7483744

[9] Easterbrook MJ, Kuppens T, Manstead ASR. The education effect: higher educational qualifications are robustly associated with beneficial personal and socio-political outcomes. Soc Indic Res. 2016;126(3):1261-1298. doi:10.1007/s11205-015-0946-1

[10] Ruijsbroek A, Wijga AH, Gehring U, Kerkhof M, Droomers M. School performance: a matter of health or socio-economic background? Findings from the PIAMA Birth Cohort Study. PLoS One. 2015;10(8):e0134780. doi:10.1371/journal.pone.0134780 26247468 PMC4527686

[11] Smirnova J, von Kobyletzki LB, Lindberg M, Svensson Å, Langan SM, Montgomery S. Atopic dermatitis, educational attainment and psychological functioning: a national cohort study. Br J Dermatol. 2019;180(3):559-564. doi:10.1111/bjd.17330 30339272 PMC7610563

[12] Rosen ML, Sheridan MA, Sambrook KA, Meltzoff AN, McLaughlin KA. Socioeconomic disparities in academic achievement: a multi-modal investigation of neural mechanisms in children and adolescents. Neuroimage. 2018;173:298-310. doi:10.1016/j.neuroimage.2018.02.043 29486324 PMC5944356

[13] Schmidt M, Schmidt SAJ, Adelborg K, . The Danish health care system and epidemiological research: from health care contacts to database records. Clin Epidemiol. 2019;11:563-591. doi:10.2147/CLEP.S179083 31372058 PMC6634267

[14] Ministry of Higher Education and Science. The Danish Education System. Published September 15, 2016. Accessed December 15, 2020. https://ufm.dk/en/publications/2016/the-danish-education-system

[15] Schmidt M, Schmidt SAJ, Sandegaard JL, Ehrenstein V, Pedersen L, Sørensen HT. The Danish National Patient Registry: a review of content, data quality, and research potential. Clin Epidemiol. 2015;7:449-490. doi:10.2147/CLEP.S91125 26604824 PMC4655913

[16] Mors O, Perto GP, Mortensen PB. The Danish Psychiatric Central Research Register. Scand J Public Health. 2011;39(7)(suppl):54-57. doi:10.1177/1403494810395825 21775352

[17] Schmidt M, Pedersen L, Sørensen HT. The Danish Civil Registration System as a tool in epidemiology. Eur J Epidemiol. 2014;29(8):541-549. doi:10.1007/s10654-014-9930-3 24965263

[18] Pottegård A, Schmidt SAJ, Wallach-Kildemoes H, Sørensen HT, Hallas J, Schmidt M. Data resource profile: the Danish National Prescription Registry. Int J Epidemiol. 2017;46(3):798-798f. doi:10.1093/ije/dyw213 27789670 PMC5837522

[19] Bliddal M, Broe A, Pottegård A, Olsen J, Langhoff-Roos J. The Danish Medical Birth Register. Eur J Epidemiol. 2018;33(1):27-36. doi:10.1007/s10654-018-0356-1 29349587

[20] Jensen VM, Rasmussen AW. Danish education registers. Scand J Public Health. 2011;39(7)(suppl):91-94. doi:10.1177/1403494810394715 21775362

[21] Baadsgaard M, Quitzau J. Danish registers on personal income and transfer payments. Scand J Public Health. 2011;39(7)(suppl):103-105. doi:10.1177/1403494811405098 21775365

[22] Heide-Jørgensen U, Adelborg K, Kahlert J, Sørensen HT, Pedersen L. Sampling strategies for selecting general population comparison cohorts. Clin Epidemiol. 2018;10:1325-1337. doi:10.2147/CLEP.S164456 30310326 PMC6165733

[23] Frisell T, Öberg S, Kuja-Halkola R, Sjölander A. Sibling comparison designs: bias from non-shared confounders and measurement error. Epidemiology. 2012;23(5):713-720. doi:10.1097/EDE.0b013e31825fa230 22781362

[24] Sjölander A, Frisell T, Kuja-Halkola R, Öberg S, Zetterqvist J. Carryover effects in sibling comparison designs. Epidemiology. 2016;27(6):852-858. doi:10.1097/EDE.0000000000000541 27488059

[25] Ministry of Higher Education and Science. Description of education in Denmark. Updated July 28, 2020. Accessed December 15, 2020. https://ufm.dk/en/education/higher-education/degrees-and-qualifications

[26] UNESCO Institute for Statistics. International Standard Classification of Education (ISCED) 2011. Accessed December 15, 2020. uis.unesco.org/sites/default/files/documents/international-standard-classification-of-education-isced-2011-en.pdf

[27] Cummings P. The relative merits of risk ratios and odds ratios. Arch Pediatr Adolesc Med. 2009;163(5):438-445. doi:10.1001/archpediatrics.2009.31 19414690

[28] Schmidt SAJ, Olsen M, Schmidt M, . Atopic dermatitis and risk of atrial fibrillation or flutter: a 35-year follow-up study. J Am Acad Dermatol. 2020;83(6):1616-1624. doi:10.1016/j.jaad.2019.08.039 31442537 PMC7704103

[29] Mitchell AE, Fraser JA, Morawska A, Ramsbotham J, Yates P. Parenting and childhood atopic dermatitis: a cross-sectional study of relationships between parenting behaviour, skin care management, and disease severity in young children. Int J Nurs Stud. 2016;64:72-85. doi:10.1016/j.ijnurstu.2016.09.016 27693983

[30] Ben-Gashir MA, Seed PT, Hay RJ. Are quality of family life and disease severity related in childhood atopic dermatitis? J Eur Acad Dermatol Venereol. 2002;16(5):455-462. doi:10.1046/j.1468-3083.2002.00495.x 12428837

[31] Jacobsen KF. The education level for the population [in Danish]. Statistics Denmark. Published October 2004. Accessed December 15, 2020. http://www.dst.dk/pukora/epub/upload/8310/befudd.pdf

