# Supplementary material for: Association Between Atopic Dermatitis and Educational Attainment in Denmark
Source: JAMA Dermatol. 2021 Apr 14;157(6):1–9. doi: 10.1001/jamadermatol.2021.0009 (PMC8047754; doi:10.1001/jamadermatol.2021.0009)
Supplement: Supplement 1. — Trial Protocol. [file jamadermatol-e210009-s001.pdf]

Study title: Hospital-diagnosed atopic eczema and educational attainment

Objectives, Specific Aims and Rationale

To examine if atopic eczema is associated with educational achievement.

A directed acyclic graph for the study is shown in the figure.

Figure showing the directed acyclic graph for the study

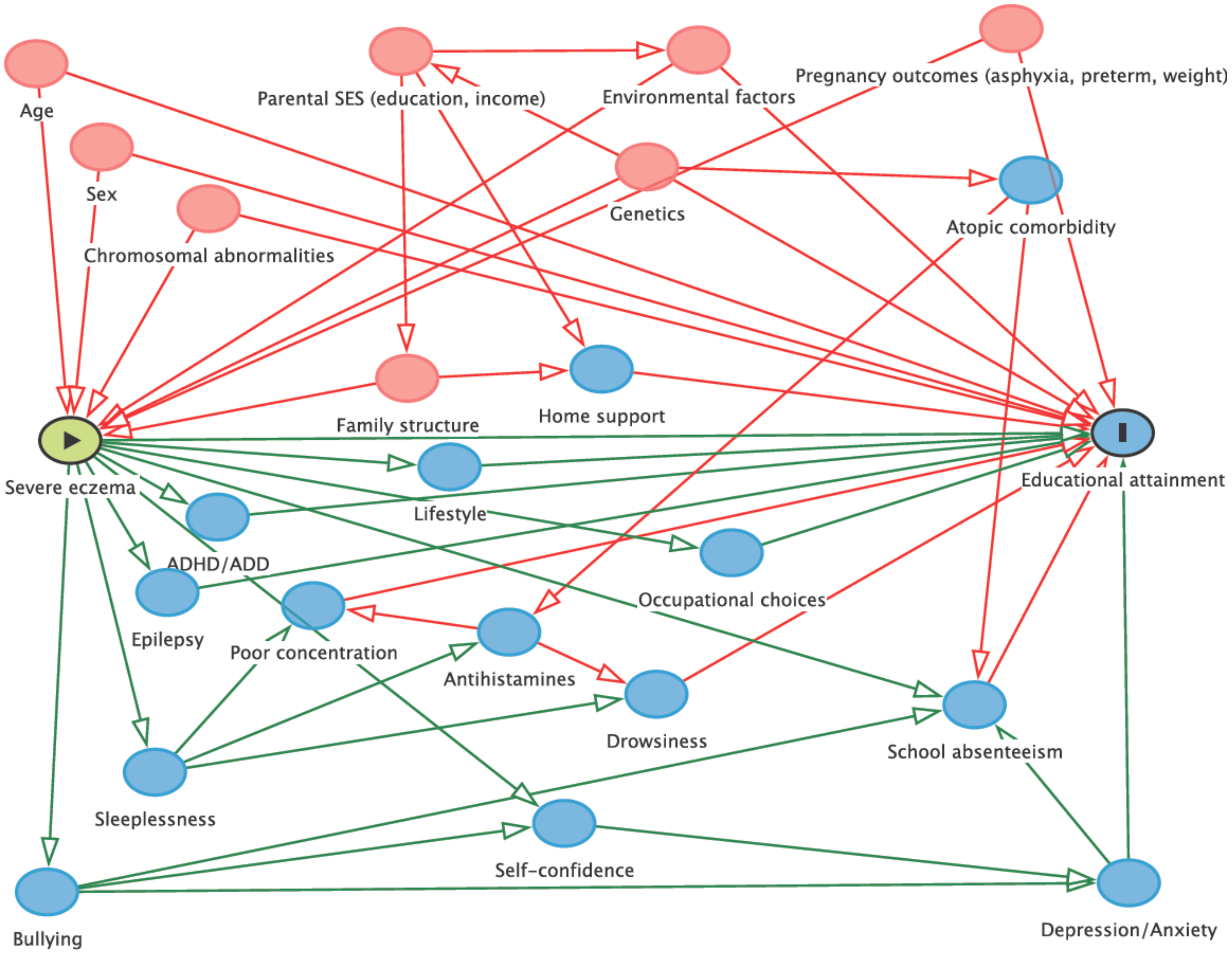

Note: Genetics illustrates various traits, including atopy (and thus risk of eczema) and e.g. intelligence. Atopic comorbidity includes allergic rhinitis and asthma.

Study Design: Nationwide cohort study.

Data sources

- The Danish National Patient Registry[1] (inpatients at non-psychiatric wards since 1977 and since 1995 admissions to psychiatric wards, visits to all outpatient hospital-based specialty clinics and emergency rooms)
- The Danish Psychiatric Central Research Registry[2] (admissions to psychiatric wards 1970–1994)
- The Civil Registration System[3] (since 1968)

The Danish National Prescription Registry[4] (since 1995)

- The Danish Medical Birth Registry[5] (since 1973)
- Socioeconomic data[6] (educational data since 1981, income data since 1980)

Study Population

The study will include an exposed cohort of persons with atopic eczema and two comparison cohorts with matched persons from the general population and full-siblings, respectively. The Danish Health Data Authority has already performed the initial sampling of these cohorts, which are described in detail below.

Eczema exposed

The Danish Health Data Authority has sampled an exposed cohort, using the following criteria:

- Persons with a diagnosis of atopic eczema (ICD-8: 691; ICD-10: DL20) in the Danish National Patient Registry between 1 Jan 1977 and 10 February 2018.
- We include all primary and secondary diagnoses for eczema from admissions, hospital outpatient clinics and emergency rooms in the Danish National Patient Registry, including ongoing contacts (that is, persons who at the end of study period are in active follow-up in a hospital outpatient clinic).
- We will consider the earliest hospital record for atopic eczema (date of admission or first outpatient appointment) to be the eczema diagnosis date.
- Only persons who are born in Denmark and living in Denmark on the eczema diagnosis date.

For the current study, we will restrict the study population defined by the Danish Health Data Authority as above further as follows:

- We will exclude eczema patients (together with their matched comparators) included by means of an ICD-8 diagnosis recorded together with an additional (“modification”) code indicating that it potentially an uncertain/unverified/working diagnosis (c_diagmod=1–7).
- We will exclude patients (together with their matched comparators) with inconsistencies in registration of status and residence in the Civil Registration System (recorded as alive but not living in Denmark but without an emigration date), as we assume that these patients are not living in Denmark and thus not eligible.
- We will limit to patients who are diagnosed with eczema prior to age 13 years and are born on 30 June 1987 or earlier. These eligibility criteria will ensure a minimum possible attained age of 30 years at end of follow-up (30 June 2017). In a sensitivity analysis, we will require that persons are born on 30 June 1982 or earlier, to ensure a higher minimum attained age (35 years) to account for the fact that some persons may not finish their final education until after age 30 years. If we experience problems with power in the main analysis, we will change the requirement for the highest possible attained age to 26 year.

Unexposed general population cohort (for main analysis)

The Danish Health Data Authority has sampled an unexposed general population cohort, as follows:

- Up to 10 persons matched to each eczema patient by sex and birth year.
- Comparators have to: (1) be born in Denmark, (2) be alive and living in Denmark on the eczema diagnosis date of their matched eczema-exposed individual, and (3) have no previous diagnosis of eczema.
- A person with atopic eczema can be included in the comparison cohort until he/she is diagnosed with eczema (and included in the exposed cohort).

As with eczema patients, we will additionally apply the following restrictions:

We will exclude matched comparators with inconsistencies in registration of status and residence in the Civil Registration System (recorded as alive but not living in Denmark but without an emigration date).

Persons in the atopic eczema cohort are eligible to be selected as comparators from birth until diagnosis of eczema. If a person from the comparison cohort is diagnosed with eczema during

follow-up (and fulfills all other eligibility criteria), he/she will also start contributing to the eczema cohort together with his/her own comparators (this has been accounted for in the sampling done by the Danish Health Data Authority).

Sibling cohort (for secondary analysis)

The comparison with the unexposed general population cohort may be associated with confounding by family-related factors, such as parental income, parental educational level, family structure, and genetic and early environmental factors. In order to adjust for stable familial characteristics, we will perform a secondary analysis making all comparisons within pairs of differentially exposed full siblings (i.e., exposure-discordant full siblings). The analysis controls for all confounders and mediators that are shared in the family (i.e., estimates the direct effect of the association between eczema and educational attainment). In this analysis, we assume that such family-related factors are more commonly shared by siblings than diagnosis of atopic eczema.[7] We also assume that the exposure and outcome of a person does not affect the exposure and outcome of the sibling (i.e., absence of 'sibling carryover'). [8]

Using the Civil Registrations System, the Danish Health Data Authority has already identified a sibling cohort consisting of all persons with the same mother and/or father as eczema patients (that is, half- and full-siblings). For this study, we will restrict this sibling cohort to full-siblings to exposed persons.

We will follow the sibling(s) from age 13 years and restrict to those without a hospital record of eczema by this age. As with eczema patients, we will also exclude siblings with inconsistencies in registration of status and residence in the Civil Registration System (recorded as alive but not living in Denmark but without an emigration date).

As atopic eczema aggregates within families, there is a greater risk of misclassification of eczema as non-eczema in the sibling analysis. In a sensitivity analysis, we will therefore also require that siblings have no prior prescription record of a topical steroid/calcineurin inhibitor (Prescription Registry ATC codes: "D07" "D11AH01" "D11AH02") at baseline. As prescription data are available only since 1995, we expect incomplete records and therefore this is not the main analysis.

Outcome definitions and follow-up

We will follow the cohort to determine the highest level of educational achievement by age 30 years. The final date of follow-up is 30 June 2017. Data on highest completed education is registered in the Population Education Registry at Statistics Denmark using the variable 'HFAUDD' (in the data table 'UDDF') which is updated annually on October 1st. The time for the achievement of the highest completed education is recorded in HF_VFRA. The HFAUDD variable is a 4-digit code for the qualification that the education leads to. We only include educations that result in a qualification (*e.g.*, finishing basic schooling). The HFAUDD-variable in itself is unsystematic and not suitable for statistics. However, it can be converted to other classification systems, including Statistics Denmark's Danish nomenclature for education ('forspalte1' until March 1, 2015 and DISCED-15 thereafter) and the International Standard Classification of Education (ISCED, currently version 2011).

The DISCED-15 was introduced on March 1, 2015 and replaced the previously used 'forsplate1' nomenclature at Statistics Denmark. DISCED-15 organizes education programs/activities in four dimensions (main area; type of education; education level (variable NIVEAU); and subject area). The main area dimension follows that of the Danish education system. [9] The first two digits specifies the overall group of education programs, e.g. basic schooling. It has no association with the ISCED. Education type has a similar structure.

The ISCED[10] was developed in 1976 by UNESCO with the aim to perform international comparisons within education. It was revised in 1997 and 2011. The structure of ISCED is developed in a collaboration between UNESCO, OECD, EU and all member states. It consists of a 7-digit code, where the first level digit describes the level/education level.

In our study, we defined study categories of the highest completed education based on the main groups of the Danish nomenclature for education. These main areas correspond largely to levels of ISCED-2011 with some minor differences for included subgroups, namely that: (1) We include information on type of youth education and (2) Medium cycle higher education programs in Denmark include "Professional bachelor's degrees" which internationally are classified as ISCED level 6 ('Bachelor or equivalent') together with Univeristy Bachelor's degree. The table below shows the categories for the current study, including registry codes, corresponding ISCED-2011 levels, a text description with examples of the educational activities/programs and jobs that it can lead to, and approximate length of education.

For the analysis, we will generate a number of binary/dummy variables (one for each education level) coded as “1” for those *not* achieving/failing to achieve that specific education level and “0” for those who have achieved the given level.

Each main educational level is conditional on completing preceding levels. That is, youth education is conditional on completing basic schooling and higher education is conditional on youth education. To account for this and for changes in covariables over time, we will define three cohorts for each of the main education levels: (1) “*Main cohort*”: For lower secondary education as the outcome, we used the main cohort of patients with AD and comparators described above and baseline date was the 13th birthday; (2) “*Lower secondary education cohort*”: For upper secondary education as the outcome, we restricted the study sample to include only those from the main cohort who attained lower secondary education prior to or at age 30 years and baseline was the time for graduation from upper secondary school; (3) “*Upper secondary education cohort*”: For higher education as the outcome, we restricted the sample further to include those who attained upper secondary education prior to or at age 30 years and baseline was the time for graduation from upper secondary school. Covariables will be redefined at baseline for each cohort. Comparators who had eczema diagnosed before or on the baseline for each cohort were excluded. In the sibling analysis, we will have corresponding three cohorts and will only include those who have no record of eczema (diagnosis or treatment) at baseline for each cohort.

We will presume that no record of a given education level means that the specific level has not been achieved. Thus, if none of the education levels are recorded, we will assume they have not finished lower secondary school (*i.e.,* basic schooling). We will exclude persons with non-consecutive recording of education, e.g. those who have lower secondary and higher education but not upper secondary education recorded, as we presume they have missing data and thus cannot follow them complete for the outcomes. A minor proportion of persons also may have died or emigrated by age 30 years and these persons will be classified with the highest education level achieved at the time of loss to follow-up.

Definition of educational achievement

Education level achieved	AFSP1H code (until February 28, 2015)	Main area of DISCED-15 (variable HOVEDOMRAADE _OVER) available from March 1, 2015	ISCED-2011 level	Description of education activities/programs, degrees, and jobs	Approximate length of education
**Lower secondary education***	"10"	"10"	2 (Lower secondary education)	Lower secondary education, which is compulsory in Denmark.	9-10 years there is an optional 10th year)
**Upper secondary education**	"20" "25" "30" "35" "39"	"20" "25" "30" "35" "39"	3 (Upper secondary education)		2–4 years
- General	"20" "25"	"20" "25"	3 (Upper secondary education)	Education programs, which primarily prepare for higher education. There are four overall programs (general, technical, commercial and preparatory).	
- Vocational	"30" "35" "39"	"30" "35" "39"	3 (Upper secondary education)	Vocational education and training, which primarily prepare for a career in a specific trade or industry. Leads to jobs like skilled craftsman, legal secretary, service function in business and trade, assistant social worker, assistant nurse, waiter, baker, cook, hairdresser.	
**Higher (tertiary) education**	"40" "50" "60" "65" "70"	"40" "50" "60" "70" "80"	5–8		
- *Short cycle*	"40"	"40"	5 (Short cycle tertiary education)	Short-cycle higher education includes mainly Academy Progression programs (in Danish: erhvervsakademiuddannelser), which are taught at business academies (prev. academy of professional higher education). These programs lead to a Academy Profession degree with the academic title "AP Graduate in …" (in Danish "AK"). The programs are typically practically-based, occupationally-specific and prepare for labor market entry. Examples of jobs that the programs may lead to are: laboratory technician, computer specialist, building technician, multimedia designer, mapping and landsurvey technician, or financial economist.	2-2.5 years
- *Medium cycle*	"50"	"50"	6 (Bachelor or	Medium cycle higher education programs are	3-4 years

			equivalent)	taught at business academies and university colleges. Many lead to a so-called professional bachelor’s degree (in Danish “Professionsbachelor”) with the title “Bachelor of/in…” (in Danish “Professionsbachelor I”/”prof.bach.”). It is considered a non-academic Bachelor degree (unlike the bachelor university degree – see long-cycle higher education). The programs are applied programs that are development-based and put special emphasis on combining theoretical studies with a practical approach. The degree can lead to jobs such as nurse, primary and lower secondary school teacher, physiotherapist, nurse, midwife, social worker, public administration, journalist, and certain types of engineers.	
- *Long cycle*	“60” “65” “70”	“60” “70” “80”	6 (Bachelor or equivalent); 7 (Master or equivalent); 8 (Doctoral or equivalent)	Long-cycle higher education include programs/activities taught at universities leading to bachelor’s degree (i.e., not “professional bachelor’s degree – see medium-cycle higher education), Master’s degree, Doctoral degree (e.g. PhD) or equivalents. Leads to jobs such as architect, civil engineer, attorney, physician, dentist, pharmacist, psychologist, theologian, anthropologist, jobs in political science and literature.	3–9 years (3 years for a Bachelor degree, approx.. 2-3 years for a Master’s degree or equivalent, and 3 years for Doctoral degree)

Notes: The ‘AFSP1H’ and ‘DISCED-15’ variables at Statistics Denmark are generated by conversion of the variable HFAUDD from the table UDDF. The time for the achievement of the highest completed education at a point is recorded in HF_VFRA.

*The HFAUDD variable identified whether an educational level results in a qualification. However, there is one exception, as 6th grade and above are included for lower secondary education regardless of whether a person graduated from lower secondary education. We will account for this by excluding the following HFAUDD codes within the group with AFSP1H/DISCED15 code “10” (includes codes for 8th grade or lower and “realskole”, a specific type of secondary school, which was abolished in 1975-78 and thus not relevant for our cohort): “1” “200” “205” “1006“ “1007“ “1008“ “1021“ “1022“ “1023“ “1100“ “1101“ “1102“ “1103“ “1104“ “1105“ “1106“ “1107“ “1108“ “1120“ “1121“ “1122“ “1123“ “1206“ “1207“ “1208“ “1410“ “1423“ “1509“ “1510“ “1522“ “1523“ “1721“ “1722“ “1723“ “2508“ ”9602“ “9603“ 9604“ “9606“ “9607“

This should leave the following HFAUDD within the group with AFSP1H/DISCED15 code “10”: “210” “1009“ “1010“ “1011“ “1109“ “1110“ “1111“ “1209“ “1210“ “2401“ “2509“ “2510“ “2511“

Secondary analysis incorporating age in the education level

With the main outcome definition, we examine if a person has achieved a certain educational level at age 30 years. This definition may not take into account that some persons may complete a specific education level but need more time to do so (i.e., the rate of achievement is lower). In a secondary analysis, we will aim to look into this in more detail by analyzing prevalence of each education level by age for eczema exposed and the comparison cohort.

Covariables

We will define various variables for the main and sensitivity analyses, as shown in the following table. Variables are defined at baseline for each cohort (except age and calendar period at atopic eczema diagnosis)

Covariables

	**Codes**	**Categorization**
Age at eczema diagnosis		For subgroup analyses: 0–5 years; 6–12 years
Sex		Female<br>Male
Calendar period at eczema diagnosis		For descriptives: 1976–1980, 1981–1985, 1986–1990, 1991–1995, 1996–2000
Attention deficit hyperactivity disorder (ADHD) / Attention deficit disorder (ADD)*	ICD-10: DF900 in the Patient Registry or The Psychiatric Central Research Registry<br>ATC: “N06BA” in the Prescription Registry	Yes, if any of the relevant codes<br>No otherwise
Depression*	ICD-8: ”29609” ”29629” ”29699” ”29809” ”30049” ”30019” in the Patient Registry or The Psychiatric Central Research Registry;<br>ICD-10: ICD-10: “DF32” ”DF33” ”DF920” in the Patient Registry or The Psychiatric Central Research Registry	Yes, if any of the relevant codes<br>No otherwise
Anxiety disorder (including phobic disorders)*	ICD-8: ”30009” ”30029”<br>in the Patient Registry or The Psychiatric Central Research Registry;<br>ICD-10: ” DF40” ”DF41” ”DF931” ”DF932” ”DF9380”<br>in the Patient Registry or The Psychiatric Central Research Registry	Yes, if any of the relevant codes<br>No otherwise
Epilepsy	ICD-8: “345” or<br>ICD-10: “DG40” “DG41” in	Yes, if any of the relevant codes<br>No otherwise

	the Patient Registry	
Asthma	ICD-8: “493” in the Danish National Patient Registry<br>ICD-10: “DJ45” in the Patient Registry<br>ATC: “R03” in the Prescription Registry	Yes, if any of the diagnosis codes or two prescriptions for drugs against obstructive lung disease bronchodilators<br>No otherwise
Rhinitis	ICD-8: “507”;<br>ICD-10: “DJ30”<br>in the Patient Registry	Yes, if any of the relevant codes<br>No otherwise
Preterm birth (<37 gestational wk)	v_svlangde (table ’t_lfoed’), Gestationsalder_dage (table ’MFR’), or Gestationsalder_uger (table ‘Hjemmefoedsler_blanket’)	Yes, if v_svlangde, Gestationsalder_uger, or Gestationsalder_dage/**7** is <37<br>No otherwise
Low birth weight (<2500 g)	V_vagt (table ’t_lfoed’), vaegt_barn (table ’MFR’), or vaegt_barn (table ‘Hjemmefoedsler_blanket’) in Medical Birth Registry	Yes, if any of variables with value <2500 g<br>No otherwise
5-min Apgar score <7 or intrauterine/birth asphyxia	V_apgar5<br>(table ‘t_lfoed’), Apgarscore_efter5minutter (table ’MFR’), or Apgarscore_efter5minutter (table ‘Hjemmefoedsler_blanket’) in Birth Registry; ICD-8 code “776” or ICD-10 code “DP20” or “DP21” in Patient Registry	Yes, if V_apgar5 or Apgarscore_efter5minutter <5 or any of the ICD-8/10 codes.<br>No otherwise
Chromosomal abnormalities (as defined by Eurocat standard code list at the department)	ICD-8 “7593” “7594” “7595”;<br>ICD-10: “DQ9”<br>in the Patient Registry	Yes, if any of the relevant codes.<br>No otherwise

Note: All subcodes are included unless otherwise stated; all types of contacts (inpatient, outpatient and emergency) and both primary and secondary diagnoses should be considered. Use admission/prescription/record date for all variables.
*Based on prelimintary analyses, variables ADHD, depression and anxiety disorder will be combined due to small numbers.

Milestones

- Statistical analyses: Preliminary results by end of March 2020; allowing for further updates/adjustments in April and May
- First version of the manuscript: Beginning of June 2020
- Submission: Beginning of July 2020

Statistical analyses

Main analyses

1. Complete the construction of the study populations, that is, apply restrictions in addition to those defined by the Danish Data Health Authority already (see section about Study Population) and define the three cohorts. We will note the number of persons excluded at each stage in this process, so that we can make a study flowchart. Also, we will look at basic descriptive statistics (calendar period, age, and sex and index date) for persons who are excluded because they have non-consecutive records of education.

2. We will compute summary statistics for covariables (at baseline for each cohort) for exposed (eczema) patients and comparison cohorts. This will make Table 1 for the study.

Table 1. Selected characteristics of eczema patients and matched comparison cohort, Denmark

	1: Main cohort		2: Lower secondary education cohort		3: Upper secondary education cohort	
	Eczema patients	Comparison cohort	Eczema patients	Comparison cohort	Eczema patients	Comparison cohort
	No. (%)	No. (%)	No. (%)	No. (%)	No. (%)	No. (%)
Total						
Age at eczema diagnosis, years						
Range						
Median (interquartile range)						
Sex						
Female						
Male						
Calendar period for eczema diagnosis (or corresponding date for comparators)						
1976–1980						
1981–1985						
1986–1990						
1991–1995						
1996–2000						
Birth year						
1973–1977						
1978–1982						
1983–1987						
Setting for first atopic dermatitis diagnosis						
Inpatient						
Outpatient hospital clinic						
Emergency room						
Attention deficit hyperactivity disorder						
Depression						
Anxiety disorder						
Asthma						
Rhinitis						
Preterm birth (<37 gestational week)						
Low birth weight (<2500 g)						
5-min Apgar score <7 or intrauterine/birth asphyxia						
Chromosomal abnormalities						

3. We will examine how many persons are lost to follow-up by age 30 years overall and for eczema patients and comparison cohorts. This will make table 2.

Table 2. Frequency of missing data on outcome and loss to follow-up of eczema patients and matched comparison cohort, Denmark. Main cohort (cohort 1)

	Eczema patients	Comparison cohort	Total
	No. (%)	No. (%)	No. (%)
Lost to follow-up by age 30 years			
Reason for loss to follow-up			
- Death			
- Emigration			
- Other			

4. For each outcome (each education level), we will estimate the number of persons not achieving the outcome, the number contributing to the analysis ('at risk'), and the prevalence at age 30 years. We will use conditional Poisson regression to estimate the risk ratio (RR) with 95% confidence intervals (CIs) of not attaining each educational level among children with AD compared with the general population comparison cohort. By conditioning on matched set, the unadjusted RR accounts for matching factors (birth year, sex, calendar period of index date). These results will make Table 3.

   Regarding choice of regression model, we wanted to estimate risk ratios rather than odds ratios because the outcome was common. We originally considered using log-binomial regression rather than logistic regression.[11] However, we experienced issues with accounting properly for the matching of AD patients and general population comparison cohort in the main analysis and family in the sibling analysis. We therefore finally chose the conditional Poisson regression as our model, as recommended by Cummings.[12]

Table 3. Risk of not having achieved a certain educational level at age 30 years among patients with atopic dermatitis compared with a matched general population cohort.

Educational achievement	AD cohort			Comparison cohort			Unadjusted risk ratio	Adjusted risk ratio
	No. not	No.	Prevalence,	No. not	No.	Prevalence,		

	achieving specific level	at risk	%	achieving specific level	at risk	%	(95% CI)[a]	(95% CI)[b]
**Lower secondary education**								
**Upper secondary education**								
**Higher education**								

[a]Unadjusted model accounts for matching factors (birth year and gender)

[b]Adjusted additionally for age at baseline.

5. We will also present the prevalence for each education level graphically by age for the eczema cohort and the comparison cohort.

Figure 1. Prevalence of each education level by age for eczema exposed and comparators

Secondary analyses (sibling comparison)

6. We will repeat the analyses (descriptive analyses, prevalence estimates and conditional Poisson regression) using the sibling comparison cohort. We will condition on family to ensure within-family comparisons in the unadjusted model. Then we will additionally adjust for age at baseline and sex. This will make tables 4–6 and figure(s) for prevalence by age.

Table 4. Selected characteristics of eczema patients and sibling cohort, Denmark

	**1: Main cohort**		**2: Lower secondary education cohort**		**3: Upper secondary education cohort**	
	**Eczema patients**	**Siblings**	**Eczema patients**	**Siblings**	**Eczema patients**	**Siblings**
	**No. (%)**	**No. (%)**	**No. (%)**	**No. (%)**	**No. (%)**	**No. (%)**
Total						
Age at eczema diagnosis, years						
Range						
Median (interquartile range)						
Sex						
Female						
Male						
Calendar period for eczema diagnosis (or corresponding date for comparators)						
1976–1980						
1981–1985						
1986–1990						
1991–1995						
1996–2000						
Birth year						
1973–1977						
1978–1982						
1983–1987						
Setting for first atopic dermatitis diagnosis						
Inpatient						
Outpatient hospital clinic						
Emergency room						
Attention deficit hyperactivity disorder						
Depression						
Anxiety disorder						
Asthma						
Rhinitis						
Preterm birth (<37 gestational week)						
Low birth weight (<2500 g)						
5-min Apgar score <7 or intrauterine/birth asphyxia						
Chromosomal abnormalities						

Table 5. Frequency of missing data on outcome and loss to follow-up of eczema patients and sibling cohort, Denmark. Main cohort (1)

	Eczema patients	Sibling cohort	Total
	**No. (%)**	**No. (%)**	**No. (%)**
Missing data on education level by age 30 years			
Lost to follow-up by age 30 years			
Reason for loss to follow-up			
- Death			
- Emigration			
- Other			

Table 6. Risk of not having achieved a certain educational level at age 30 years among patients with atopic dermatitis compared with siblings.

**Educational achievement**	**AD cohort**			**Sibling cohort**			**Risk ratio (95% CI)**	
	**No. not achieving specific level**	**No. at risk**	**Prevalence, %**	**No. not achieving specific level**	**No. at risk**	**Prevalence, %**	**Unadjusted[a]**	**Adjusted[b]**
**Lower secondary education**								
**Upper secondary education**								
**Higher education**								
- Short cycle								
- Medium cycle								
- Long								

cycle								

[a]Unadjusted model accounts for family.

[b]Adjusted additionally for age at baseline and sex.

Figure 2. Prevalence of each education level by age for eczema exposed and siblings

Secondary analyses (subgroup/stratified analyses)

Note: Stratified and sensitivity analyses will be performed for overall education levels only.

7. We will explore whether the association between atopic dermatitis and educational achievement differ by age. This will make Tables 7a and 7b.

Table 7a. Risk of not having achieved a certain educational level at age 30 years among patients with atopic dermatitis compared with a matched general population cohort, **age 0–5 years** at eczema diagnosis (or corresponding date for comparators)

**Educational achievement**	**AD cohort**			**Comparison cohort**			**Risk ratio (95% CI)**	
	**No. not achieving specific level**	**No. at risk**	**Prevalence, %**	**No. not achieving specific level**	**No. at risk**	**Prevalence, %**	**Unadjusted[a]**	**Adjusted[b]**
**General population comparison cohort**								
Lower secondary education								
Upper secondary education								
Higher education								
**Sibling cohort**								
Lower secondary education								
Upper secondary education								
Higher								

education								

[a]Unadjusted model accounts for matching factors in the analysis with the main comparison cohort and for family in the analysis with the sibling cohort.

[b]Adjusted additionally for age at baseline and, in sibling analysis, sex.

Table 7b. Risk of not having achieved a certain educational level at age 30 years among patients with atopic dermatitis compared with a matched general population cohort, **age 6–12 years** at eczema diagnosis (or corresponding date for comparators)

**Educational achievement**	**AD cohort**			**Comparison cohort**			**Risk ratio (95% CI)**	
	**No. not achieving specific level**	**No. at risk**	**Prevalence, %**	**No. not achieving specific level**	**No. at risk**	**Prevalence, %**	**Unadjusted[a]**	**Adjusted[b]**
**General population comparison cohort**								
Lower secondary education								
Upper secondary education								
Higher education								
**Sibling cohort**								
Lower secondary education								
Upper secondary education								
Higher education								

[a]Unadjusted model accounts for matching factors in the analysis with the main comparison cohort and for family in the analysis with the sibling cohort.

[b]Adjusted additionally for age at baseline and, in sibling analysis, sex.

8. We will explore whether the association between atopic dermatitis and educational achievement differs by sex. This will make Tables 8a and 8b.

Table 8a. Risk of not having achieved a certain educational level at age 30 years among patients with atopic dermatitis compared with a matched general population cohort, **men**

**Educational achievement**	**AD cohort**			**Comparison cohort**			**Risk ratio (95% CI)**	
	**No. not achieving specific level**	**No. at risk**	**Prevalence, %**	**No. not achieving specific level**	**No. at risk**	**Prevalence, %**	**Unadjusted[a]**	**Adjusted[b]**
**General population comparison cohort**								
Lower secondary education								
Upper secondary education								
Higher education								
**Sibling cohort**								
Lower secondary education								
Upper secondary education								
Higher education								

[a]Unadjusted model accounts for matching factors in the analysis with the main comparison cohort and for family in the analysis with the sibling cohort.

[b]Adjusted additionally for age at baseline and, in sibling analysis, sex.

Table 8b. Risk of not having achieved a certain educational level at age 30 years among patients with atopic dermatitis compared with a matched general population cohort, **women**

**Educational achievement**	**AD cohort**			**Comparison cohort**			**Risk ratio (95% CI)**	
	**No. not achieving specific level**	**No. at risk**	**Prevalence, %**	**No. not achieving specific level**	**No. at risk**	**Prevalence, %**	**Unadjusted[a]**	**Adjusted[b]**
**General population comparison cohort**								
Lower secondary education								
Upper secondary education								
Higher education								
**Sibling cohort**								
Lower secondary education								
Upper secondary education								
Higher education								

[a]Unadjusted model accounts for matching factors in the analysis with the main comparison cohort and for family in the analysis with the sibling cohort.

[b]Adjusted additionally for age at baseline and, in sibling analysis, sex.

9. For the sibling comparison, we will explore whether the association between atopic dermatitis and educational attainment differ by parents income and educational level (both defined below). This will make Tables 9a and 9b.

Parental income	Categorized based on quartiles of the variable PERINDKIALT_13 (during 1987-) and PERINDKIALT (1980-1986) using the income for the parent with the highest income.
Parental educational level at index date	As defined for outcome (lower secondary education, upper secondary education, higher education) using the education level for the parent with the highest education.

Table 9a. Risk of not having achieved a certain educational level at age 30 years among patients with atopic dermatitis compared with **sibling cohort**, **by parental income**

**Educational achievement**	**AD cohort**			**Siblings**			**Risk ratio (95% CI)**	
	**No. achieving specific level**	**No. at risk**	**Prevalence, %**	**No. achieving specific level**	**No. at risk**	**Prevalence, %**	**Unadjusted[a]**	**Adjusted[b]**
**Parental income in quartile 1**								
Lower secondary education								
Upper secondary education								
Higher education								
**Parental income in quartile 2**								
Lower secondary education								
Upper								

secondary education								
Higher education								
**Parental income in quartile 3**								
Lower secondary education								
Upper secondary education								
Higher education								
**Parental income in quartile 4**								
Lower secondary education								
Upper secondary education								
Higher education								

[a]Unadjusted model accounts for family.

[b]Adjusted additionally for age at baseline and sex.

Table 9b. Risk of not having achieved a certain educational level at age 30 years among patients with atopic dermatitis compared with **sibling cohort**, **by parental educational level**

**Educational achievement**	**AD cohort**			**Siblings**			**Risk ratio (95% CI)**	
	**No. achieving specific level**	**No. at risk**	**Prevalence, %**	**No. achieving specific level**	**No. at risk**	**Prevalence, %**	**Unadjusted[a]**	**Adjusted[b]**
**Highest**								

**parental educational level: Lower secondary education**								
Lower secondary education								
Upper secondary education								
Higher education								
**Highest parental educational level: Upper secondary education**								
Lower secondary education								
Upper secondary education								
Higher education								
**Highest parental educational level: higher education**								
Lower secondary education								
Upper secondary education								

Higher education								

[a]Unadjusted model accounts for family.

[b]Adjusted additionally for age at baseline and sex.

Sensitivity analyses

10. We will examine the impact of adjusting additionally for attention deficit (hyperactivity) disorder, depression, anxiety disorder, epilepsy, asthma, and rhinitis to ensure that associations are not explained by presence of other diseases that are more common among atopic dermatitis patients due to common pathophysiology or as a consequence of atopic disease (i.e., possible mediators). This will make table 10.

Table 10. Risk of not having achieved a certain educational level at age 30 years among patients with atopic dermatitis compared with a matched general population cohort and a sibling cohort, unadjusted model, adjusted model from main analysis and a model with additional adjustment for attention deficit hyperactivity disorder, depression, anxiety disorder, asthma, and rhinitis

**Educational achievement**	**Unadjusted**[a]	**Adjusted**[b]	**Fully-adjusted**[c]
**General population comparison cohort**			
Lower secondary education			
Upper secondary education			
Higher education			
**Sibling cohort**			
Lower secondary education			
Upper secondary education			
Higher education			

[a]Unadjusted model accounts for matching factors (birth year, sex, and calendar year) in the analysis with the main comparison cohort and for family in the analysis with the sibling cohort.

[b]Adjusted for age at baseline and, in sibling analysis, sex

[c]Adjusted additionally for attention deficit hyperactivity disorder, depression, anxiety disorder, asthma, and rhinitis

11. We will repeat analyses after restricting further to patients who were born on 30 June 1982 or earlier to be able to determine outcome at age 35 years instead of 30 years. This will make table 11.

Table 11. Educational achievement among patients with atopic dermatitis compared with a matched general population and sibling cohort, restricting to those diagnosed prior to age 13 years in the period 1 January 1981 to 1 January 1995 to ensure a minimum attained age of 35 years.

**Educational achievement**	**AD cohort**			**Comparison cohort**			**Risk ratio (95% CI)**	
	**No. not achieving specific level**	**No. at risk**	**Prevalence, %**	**No. not achieving specific level**	**No. at risk**	**Prevalence, %**	**Unadjusted[a]**	**Adjusted[b]**
**General population comparison cohort**								
Lower secondary education								
Upper secondary education								
Higher education								
**Sibling cohort**								
Lower secondary education								
Upper secondary education								
Higher education								

[a]Unadjusted model accounts for matching factors in the analysis with the main comparison cohort and for family in the analysis with the sibling cohort.

[b]Adjusted for age at baseline and, in sibling analysis, sex

12. We will repeat analyses after excluding individuals in both cohorts who were born preterm, who had a 5-min Apgar score <7 or intrauterine/birth asphyxia, who had low birth weight or chromosomal abnormalities. This will make table 12.

Table 12. Educational achievement among patients with atopic dermatitis compared with a matched general population and sibling cohort, sensitivity analysis excluding who had a 5-min Apgar score <7 or intrauterine/birth asphyxia, who had low birth weight or chromosomal abnormalities.

**Educational achievement**	**AD cohort**	**Comparison cohort**	**Risk ratio**

							(95% CI)	
	**No. not achieving specific level**	**No. at risk**	**Prevalence, %**	**No. not achieving specific level**	**No. at risk**	**Prevalence, %**	**Unadjusted[a]**	**Adjusted[b]**
**General population comparison cohort**								
Lower secondary education								
Upper secondary education								
Higher education								
**Sibling cohort**								
Lower secondary education								
Upper secondary education								
Higher education								

[a]Unadjusted model accounts for matching factors in the analysis with the main comparison cohort and for family in the analysis with the sibling cohort.

[b]Adjusted for age at baseline and, in sibling analysis, sex

Sensitivity analyses pertaining to the sibling design specifically

13. We will repeat the main analysis based on the subset of eczema patients (and their comparators) who are also included in the sibling analyses (*i.e.*, eczema patients who had at least one sibling in the dataset), in order to compare the results from the analysis for the matched general comparison cohort and the sibling cohort. This analysis examines the assumption that results for sibling comparisons will generalize to other samples (e.g., families with only one child, families without variability in the outcome). I.e., to ensure that any difference between main and sibling comparisons, is not due to the exclusion of single-offspring families. This will make Table 13.

Table 13. Educational achievement among patients with atopic dermatitis compared with a matched general population cohort, restricting only to eczema patients (and their comparators) who are also included in the sibling analyses

Educational achievement	AD cohort			Comparison cohort			Unadjusted risk ratio (95% CI)[a]	Adjusted risk ratio (95% CI)[b]
	No. not achieving specific level	No. at risk	Prevalence, %	No. not achieving specific level	No. at risk	Prevalence, %		
**Lower secondary education**								
**Upper secondary education**								
**Higher education**								

[a]Unadjusted model accounts for matching factors (birth year and gender)

[b]Adjusted additionally for age at baseline.

14. The sibling design makes it possible to adjust for confounding by measured and unmeasured family-related factors, which are stable. However, some factors of interest, e.g., parental income, parental educational level, and family structure (living with single parent), may possible change over time and differ between siblings. In order to address this potential limitation, we will repeat the analysis for siblings with an age difference of 3 years or less. This also limits any confounding by differences in calendar period (in case there are any political changes with impact on educational attainment during the study). This will make Table 14.

Table 14. Educational achievement among patients with atopic dermatitis compared with a sibling cohort, restricting to siblings with an age difference of 3 years or less

Educational achievement	AD cohort			Sibling cohort			Risk ratio (95% CI)	
	No. not achieving specific level	No. at risk	Prevalence, %	No. not achieving specific level	No. at risk	Prevalence, %	Unadjusted[a]	Adjusted[b]
Lower secondary education								
Upper secondary education								
Higher education								

[a]Unadjusted model accounts for matching factors in the analysis with the main comparison cohort and for family in the analysis with the sibling cohort.

[b]Adjusted for age at baseline and sex

15. As atopic eczema aggregates within families, there is a greater risk of misclassification of eczema as non-eczema in the sibling analysis. We will therefore do a sensitivity analysis where we also require that siblings have no prior prescription record (available since 1995) of a topical steroid/calcineurin inhibitor (Prescription Registry ATC codes: “D07” “D11AH01” “D11AH02”) at baseline. This will make table 15.

Table 15. Educational achievement among patients with atopic dermatitis compared with a sibling cohort, excluding siblings with prescription records of topical steroid/calcineurin inhibitors at baseline (suggestive of eczema)

Educational achievement	AD cohort			Sibling cohort			Risk ratio (95% CI)	
	No. not	No. at risk	Prevalence,	No. not	No. at risk	Prevalence,	Unadjusted[a]	Adjusted[b]

	**achieving specific level**		**%**	**achieving specific level**		**%**		
**Lower secondary education**								
**Upper secondary education**								
**Higher education**								

[a]Unadjusted model accounts for matching factors in the analysis with the main comparison cohort and for family in the analysis with the sibling cohort.

[b]Adjusted for age at baseline and sex

16. One assumption for the sibling comparison is that the family-related factors that we wish to control for are more commonly shared by siblings (i.e., more strongly correlated) than diagnosis of atopic eczema. This assumption cannot be verified completely, but we will produce some descriptive statistics showing the correlation of parental income (see definition above) and parental educational level (see definition above), respectively, among siblings. Correlation of AD in families will be sought in the literature for comparison.

Amendment on July 6, 2020

The original protocol included a secondary analysis where we aimed to compute the prevalence and relative risk of subtypes of upper secondary education and higher education. However, we accounted methodological problems with separating different subtypes of education in a meaningful way, as the reference group (outcome=0) became difficult to interpret. For example, the reference group for

examining the risk of not attaining vocational education would be a mix of persons who do not attain any type of upper secondary education and persons attaining general upper secondary education. Thus, it because difficult to tell if any association reflects a failing level of education or choosing another type of education. As we overall found no substantial association between eczema and the educational outcomes, and because adjustments had limited impact on RR, we therefore chose a simple approach where we computed the probability of subtypes of upper secondary education and higher education in children with atopic dermatitis, the matched general population comparison cohort, and cohort of full-siblings.

References

1 Schmidt M, Schmidt SAJ, Sandegaard JL, Ehrenstein V, Pedersen L, Sørensen HT. The Danish National Patient Registry: A review of content, data quality, and research potential. *Clin Epidemiol* 2015; **7**: 449–90.

2 Mors O, Perto GP, Mortensen PB. The Danish Psychiatric Central Research Register. *Scand J Public Health* 2011; **39**: 54–7.

3 Schmidt M, Pedersen L, Sørensen HT. The Danish Civil Registration System as a tool in epidemiology. *Eur J Epidemiol* 2014; **29**: 541–9.

4 Pottegård A, Schmidt SAJ, Wallach-Kildemoes H, Sørensen HT, Hallas J, Schmidt M. Data Resource Profile: The Danish National Prescription Registry. *Int J Epidemiol* 2017; **46**: 798–798f.

5 Bliddal M, Broe A, Pottegård A, Olsen J, Langhoff-Roos J. The Danish Medical Birth Register. *Eur J Epidemiol* 2018; **33**: 27–36.

6 Jensen VM, Rasmussen AW. Danish Education Registers. *Scand J Public Health* 2011; **39**: 91–4.

7 Frisell T, Öberg S, Kuja-Halkola R, Sjölander A. Sibling Comparison Designs. *Epidemiology* 2012; **23**: 713–20.

8 Sjölander A, Frisell T, Kuja-Halkola R, Öberg S, Zetterqvist J. Carryover Effects in Sibling Comparison Designs. *Epidemiology* 2016; **27**: 852–8.

9 Description of Education in Denmark. Ministry of Higher Education and Science. https://ufm.dk/en/education/higher-education/degrees-and-qualifications.

10 ISCED 2011. The United Nations Educational, Scientific and Cultural Organization (UNESCO) Institute for Statistics. 2012. http://uis.unesco.org/sites/default/files/documents/international-standard-classification-of-education-isced-2011-en.pdf (accessed Jan 21, 2020).

11 McNutt L-A, Wu C, Xue X, Hafner JP. Estimating the relative risk in cohort studies and clinical trials of common outcomes. *Am J Epidemiol* 2003; **157**: 940–3.

12 Cummings P. The relative merits of risk ratios and odds ratios. *Arch Pediatr Adolesc Med* 2009; **163**: 438–45.
