# Supplementary material for: Association Between Atopic Dermatitis and Educational Attainment in Denmark
Source: JAMA Dermatol. 2021 Apr 14;157(6):1–9. doi: 10.1001/jamadermatol.2021.0009 (PMC8047754; doi:10.1001/jamadermatol.2021.0009)
Supplement: Supplement 2. — eMethods 1. Description of Study Setting and Definitions of Variables eMethods 2. Details of Sensitivity Analyses eFigure 1. Flowchart for Main Analysis eFigure 2. Flowchart for Sibling Analysis eFigure 3. Probabilities and Risk Ratios (RRs) for not Attaining Specific Educational Levels eTable 1. Probability of Subtypes of Upper Secondary Education and Higher Education eTable 2. Probability and Risk Ratios (RRs) for not Attaining Specific Educational Levels eTable 3. Correlation Between Parental Income and Parental Educational Level Among Exposure-Discordant Siblings eReferences. [file jamadermatol-e210009-s002.pdf]

Supplementary Online Content

Schmidt SAJ, Mailhac A, Darvalics B, et al. Association between atopic dermatitis and educational attainment in Denmark. *JAMA Dermatol*. Published online April 14, 2021. doi:10.1001/jamadermatol.2021.0009

eMethods 1. Description of Study Setting and Definitions of Variables
eMethods 2. Details of Sensitivity Analyses
eFigure 1. Flowchart for Main Analysis
eFigure 2. Flowchart for Sibling Analysis
eFigure 3. Probabilities and Risk Ratios (RRs) for not Attaining Specific Educational Levels
eTable 1. Probability of Subtypes of Upper Secondary Education and Higher Education
eTable 2. Probability and Risk Ratios (RRs) for not Attaining Specific Educational Levels
eTable 3. Correlation Between Parental Income and Parental Educational Level Among Exposure-Discordant Siblings
eReferences.

This supplementary material has been provided by the authors to give readers additional information about their work.

© 2021 Schmidt et al. *JAMA Dermatol*.

eMethods 1. Description of Study Setting and Definitions of Variables

Setting

The Danish welfare model is designed to promote health and social equity through universal access to various tax-financed services.[1] Thus, prepaid healthcare access is guaranteed for all residents. Some out-of-pocket expenditure is required for outpatient dispensing of prescription drugs, but the percentage of costs reimbursed increases with amount of expenditure (beginning at 60% for children). The educational system is also open to all students. Admission to all levels of education is free, except for tuition fees for private elementary schools.[2] Furthermore, student aid provides economic support to all persons aged 18 or older who are enrolled in upper secondary or higher education, regardless of socioeconomic status.[3] In 2020, pre-tax monthly aid for students in a full-degree programme of higher education was 6243 Danish kroner (≈906 US dollars). Supplementary state student loans are available.

Data sources

In Denmark, there is a long tradition for recording individual-level health and social data in various nationwide registries, using the unique personal identifiers assigned to all Danish residents since 1968. [4] We used the Danish National Patient Registry[5] and Psychiatric Central Research Registry[6] to identify data from the hospital sector, including non-psychiatric hospitals wards since 1977 and psychiatric wards and outpatient hospital-based specialty clinics and emergency rooms since 1995. The Civil Registration System provided data on demographics, death, migration, and close kinship,[4] thus allowing sampling of comparison cohorts and complete follow-up. The Danish National Prescription Registry provided information on prescription drugs dispensed at Danish pharmacies since 1995.[7] We used the Danish Medical Birth Registry, established in 1973, to obtain data on certain birth outcomes.[8] We used socioeconomic data recorded by Statistics Denmark, including educational data and income data. [9,10] Detailed definitions, including coding, of exposure, outcomes and covariables based on these data sources are provided in designated sections below. The study cohorts were sampled by the Danish Health Data Authority and transferred to secure servers at Statistics Denmark, the central authority on Danish statistics, for individual-level linkage of all registries. There, further refinement of cohorts, data cleaning and analyses were conducted by one of the coauthors (AM) on the deidentified data.

© 2021 Schmidt et al. *JAMA Dermatol.*

Exposure data

Our study exposure was a first-time hospital diagnosis of atopic dermatitis (AD) in the Danish National Patient Registry, identified by the 8th version of the International Classification of Diseases (ICD-8) code 691 during 1 January 1977 to 31 December 1993 and ICD-10 code L20 during 1 January 1994 to 30 June 2000 (latest inclusion date). The first record (admission or contact) of an AD diagnosis was taken to be the diagnosis date. We considered a diagnosis whether it was listed as a primary diagnosis or a secondary (contributing) diagnosis from an admission or contact to an outpatient clinics or emergency room. Because recording of diagnoses from outpatient clinics began in 1995, most children with AD in our study were diagnosed during an admission and likely had severe disease.

We excluded comparators with an AD diagnosis recorded in the registry on or before baseline for each main and sibling comparison cohort. Comparators who were diagnosed with AD during follow-up joined the AD cohort (together with their own comparators) at next baseline (assuming they had completed the previous education level).

Outcome data

We used the Population Education Registry at Statistics Denmark to retrieve data on completed education. The Registry is based on administrative records from education institutions and is supplemented with self-reported information for persons who completed education before 1974 and immigrants schooled outside Denmark. Data are updated annually on October 1st.

We followed the study cohorts to determine the highest level of education attained by age 30. Last collection date was 30 June 2017. We defined three main groups of education using Statistics Denmark's Danish nomenclature for education (DISCED-15), as outlined in **Table A** below. The DISCED-15 was introduced on March 1, 2015 and replaced a similar nomenclature ('forspalte1') used locally at Statistics Denmark. DISCED-15 organises education programmes/activities in four dimensions (main area; type of education; education level; and subject area). The main area dimension follows that of the Danish education system.[11] The first two digits specify the overall group of education programs, *e.g.*, basic schooling. DISCED-15 has no association with the International Standard Classification of Education, version 2011 (ISCED-2011),[2,12] but corresponding levels are shown in **Table A**.

© 2021 Schmidt et al. *JAMA Dermatol.*

Table A. Definitions of educational levels

**Education level attained**	**AFSP1H code (until February 28, 2015)**	**Main area of DISCED-15 (variable HOVEDOMRAADE _OVER) available from March 1, 2015**	**ISCED-2011 level**	**Description of education activities/programs, degrees, and jobs**	**Approximate length of education**
**Lower secondary education***	"10"	"10"	2 (Lower secondary education)	Lower secondary education, which is compulsory in Denmark.	9-10 years there is an optional 10th year)
**Upper secondary education**	"20" "25" "30" "35" "39"	"20" "25" "30" "35" "39"	3 (Upper secondary education)		2–4 years
- General	"20" "25"	"20" "25"	3 (Upper secondary education)	Education programs, which primarily prepare for higher education. There are four overall programs (general, technical, commercial and preparatory).	
- Vocational	"30" "35" "39"	"30" "35" "39"	3 (Upper secondary education)	Vocational education and training, which primarily prepare for a career in a specific trade or industry. Leads to jobs like skilled craftsman, legal secretary, service function in business and trade, assistant social worker, assistant nurse, waiter, baker, cook, hairdresser.	
**Higher (tertiary) education**	"40" "50" "60" "65" "70"	"40" "50" "60" "70" "80"	5–8		
- *Short cycle*	"40"	"40"	5 (Short cycle tertiary education)	Short-cycle higher education includes mainly Academy Progression programs (in Danish: erhvervsakademiuddannelser), which are taught at business academies (prev. academy of professional higher education). These programs lead to a Academy Profession degree with the academic title "AP Graduate in …" (in Danish "AK"). The programs are typically practically-based, occupationally-specific and prepare for labor market entry. Examples of jobs that the programs may lead to are: laboratory technician, computer specialist, building technician, multimedia designer, mapping and landsurvey technician, or financial economist.	2-2.5 years

© 2021 Schmidt et al. *JAMA Dermatol*.

- *Medium cycle*	"50"	"50"	6 (Bachelor or equivalent)	Medium cycle higher education programs are taught at business academies and university colleges. Many lead to a so-called professional bachelor's degree (in Danish "Professionsbachelor") with the title "Bachelor of/in…" (in Danish "Professionsbachelor I"/"prof.bach."). It is considered a non-academic Bachelor degree (unlike the bachelor university degree – see long-cycle higher education). The programs are applied programs that are development-based and put special emphasis on combining theoretical studies with a practical approach. The degree can lead to jobs such as nurse, primary and lower secondary school teacher, physiotherapist, nurse, midwife, social worker, public administration, journalist, and certain types of engineers.	3-4 years
- *Long cycle*	"60" "65" "70"	"60" "70" "80"	6 (Bachelor or equivalent);<br>7 (Master or equivalent);<br>8 (Doctoral or equivalent)	Long-cycle higher education include programs/activities taught at universities leading to bachelor's degree (i.e., not "professional bachelor's degree – see medium-cycle higher education), Master's degree, Doctoral degree (e.g. PhD) or equivalents. Leads to jobs such as architect, civil engineer, attorney, physician, dentist, pharmacist, psychologist, theologian, anthropologist, jobs in political science and literature.	3–9 years (3 years for a Bachelor degree, approx.. 2-3 years for a Master's degree or equivalent, and 3 years for Doctoral degree)

Notes: The 'AFSP1H' and 'DISCED-15' variables at Statistics Denmark are generated by conversion of the variable HFAUDD from the table UDDF. The time for the achievement of the highest completed education at a point in time is recorded in HF_VFRA.

*The HFAUDD variable identifies whether an educational level results in a qualification. However, there is one exception, as 6th grade and above are included for lower secondary education regardless of whether a person graduated from lower secondary education. As we only included educations that resulted in a qualification (*e.g.*, finishing lower secondary education), we accounted for this by excluding the following HFAUDD codes within the group with AFSP1H/DISCED15 code "10" (includes codes for 8th grade or lower and "realskole", a specific type of secondary school, which was abolished in 1975-78 and thus not relevant for our study):
"1" "200" "205" "1006" "1007" "1008" "1021" "1022" "1023" "1100" "1101" "1102" "1103" "1104" "1105" "1106" "1107" "1108" "1120" "1121" "1122" "1123" "1206" "1207" "1208" "1410" "1423" "1509" "1510" "1522" "1523" "1721" "1722" "1723" "2508" "9602" "9603" 9604" "9606" "9607". This leaves the following HFAUDD within the group with AFSP1H/DISCED15 code "10": "210" "1009" "1010" "1011" "1109" "1110" "1111" "1209" "1210" "2401" "2509" "2510" "2511"

© 2021 Schmidt et al. *JAMA Dermatol*.

Missing education data

In 2007, missing data on educational level was found for 3% of ethnic Danes born in 1945–1990 and up to 15% of immigrants.[9] We restricted to persons born in Denmark to limit such misclassification. We consider it unlikely that any remaining misclassification is related to having AD.

Missing data due to death or emigration is also possible. We classified persons who died or emigrated by age 30 according to the highest educational level attained at loss to follow-up. In the absence of a record for a given educational level, we assumed that it had not been attained. If no educational level was recorded, we assumed that lower secondary education had not been attained. For each study cohort, we excluded persons who had emigrated or died at baseline. As shown in **Table B** below, lost to follow-up was minor (1.1% or less for all cohorts). In particular, emigration was similar in the AD and comparison cohorts, suggesting that bias due to missing data from studies abroad is unlikely.

Table B. Frequency of loss to follow-up for children with atopic dermatitis, a matched general population comparison cohort, and a cohort of full-siblings.

	**Main analysis**						**Sibling analysis**					
	**Total**		**AD cohort**		**Comparison cohort**		**Total**		**AD cohort**		**Sibling cohort**	
**Cohort 1: Main cohort**	**N**	**%**	**N**	**%**	**N**	**%**	**N**	**%**	**N**	**%**	**N**	**%**
No loss to follow-up	60,983	99.7	5906	99.7	55,077	99.7	7283	99.7	3250	99.7	4033	99.7
Emigration	77	0.1	11	0.2	66	0.1	a	a	a	a	a	a
Death	93	0.2	10	0.2	83	0.2	a	a	a	a	a	a
**Cohort 2: Lower secondary education cohort**												
No loss to follow-up	58,252	99.3	5724	99.1	52,528	99.3	6960	99.1	3118	99.2	3842	99.1
Emigration	179	0.3	18	0.3	161	0.3	32	0.5	11	0.4	21	0.5
Death	245	0.4	35	0.6	210	0.4	29	0.4	15	0.5	14	0.4
**Cohort 3: Upper secondary education cohort**												
No loss to follow-up	39,594	98.9	4589	99.0	35,005	98.9	5003	99.0	2288	99.1	2715	98.9
Emigration	334	0.8	34	0.7	300	0.9	34	0.7	14	0.6	20	0.7
Death	116	0.3	13	0.3	103	0.3	18	0.4	7	0.3	11	0.4

Abbreviation: AD=atopic dermatitis
[a]Not shown to preserve confidentiality.

© 2021 Schmidt et al. *JAMA Dermatol.*

We encountered issues with inconsistent recording of consecutive educational levels, *e.g.* persons who had completed lower secondary and higher education but without a record of upper secondary education. Persons with such inconsistencies in the sequence of educational records were excluded, assuming that this reflected missing outcome data or loss to follow-up. However, we note that only 2293 persons (3.3% of the study sample) were excluded on this account and their characteristics were similar to those of the final study population (see **Table C** below).

Table C. Basic descriptive data for persons included in the study and those excluded due to inconsistencies in the sequence of education records.

	**Non-consecutive education records**			
	**Yes**		**No**	
	**N**	**%**	**N**	**%**
**All**	2293	100.0	67399	100.0
**Birth year**				
1973	130	5.7	2882	4.3
1974	129	5.6	3268	4.9
1975	165	7.2	4363	6.5
1976	156	6.8	4320	6.4
1977	177	7.7	4975	7.4
1978	157	6.9	4700	7.0
1979	162	7.1	4412	6.6
1980	170	7.4	4560	6.8
1981	149	6.5	4509	6.7
1982	124	5.4	4368	6.5
1983	156	6.8	4981	7.4
1984	189	8.2	5663	8.4
1985	181	7.9	5987	8.9
1986	190	8.3	6523	9.7
1987	58	2.5	1888	2.8
**Sex**				
Male	1189	51.9	37,895	56.2
Female	1104	48.2	29,504	43.8
**Calendar period of atopic dermatitis diagnosis**				
NA (comparators)	2077	90.6	61,472	91.2
1976–1980	58	2.5	1445	2.1
1981–1985	66	2.9	2010	3.0
1986–1990	76	3.3	1658	2.5
1991–2000	16	0.7	814	1.2

© 2021 Schmidt et al. *JAMA Dermatol.*

Covariables

Our proposed directed acyclic graph shown in the **Figure** below helped to identify relevant covariables. We defined variables at baseline for each cohort (except age and calendar period at AD diagnosis, which were constant). **Table D** shows the coding of covariables. As potential mediators, we included hospital diagnoses of attention deficit (hyperactivity) disorder, depression and anxiety disorders (combined as one variable because of rarity),[13-18] epilepsy,[19-21] asthma, and rhinitis. Asthma and rhinitis could also be effect modifiers (as markers of atopic severity) or confounders.[13-15,22] Although only weak links to AD have been described, [23-27] we identified birth outcomes that may affect academic performance (preterm birth, low birth weight, 5-minute Apgar score <7, intrauterine/birth asphyxia, and chromosomal abnormalities).

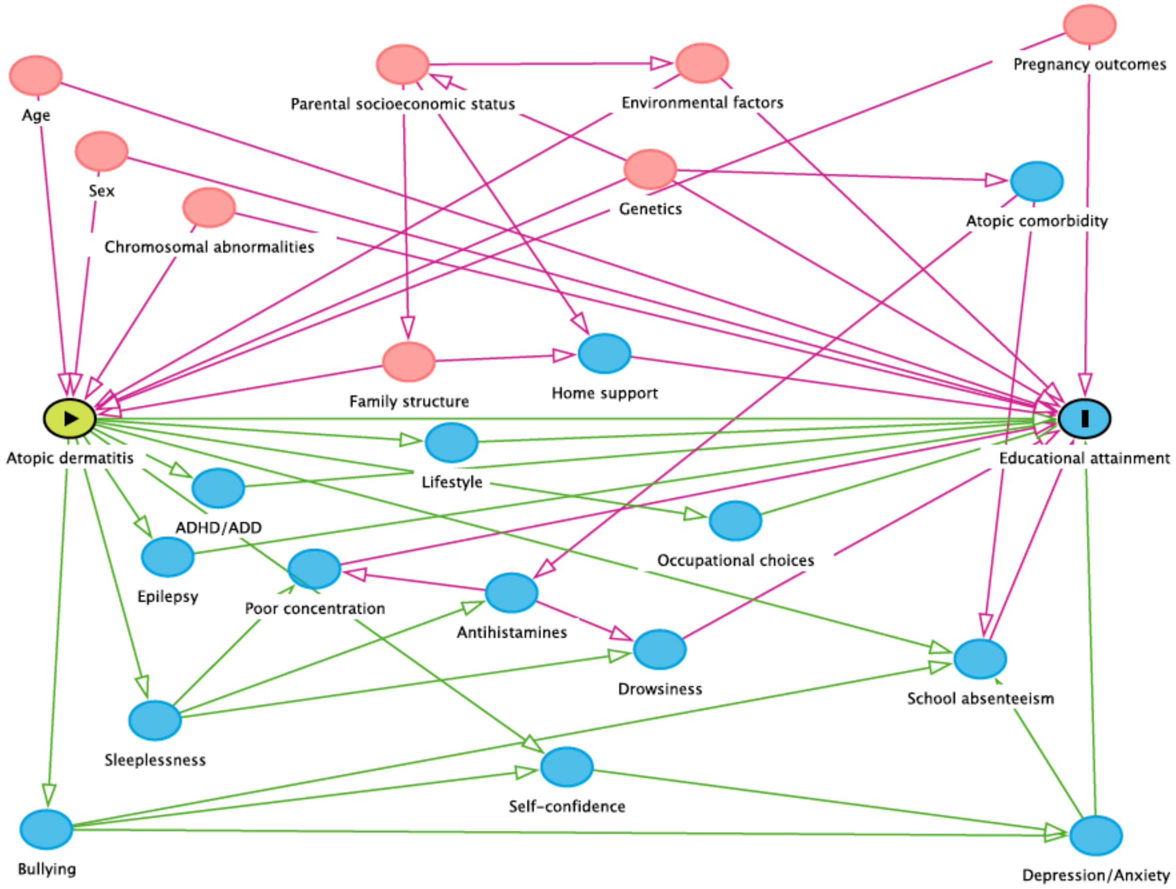

Figure. Directed acyclic graph for the study.

Notes: Genetics illustrates various traits, including risk of atopic disease atopic disease and *e.g.* intelligence. Atopic comorbidity includes asthma and allergic rhinitis.

© 2021 Schmidt et al. *JAMA Dermatol.*

Table D. Coding of covariables

	**Codes**	**Categorisation**
Age at atopic dermatitis diagnosis		Categories <5 years and 5–12 years
Sex		Female<br>Male
Calendar period of atopic dermatitis diagnosis		1977–1982, 1983–1988, 1989–1994, 1995–2000
Attention deficit (hyperactivity) disorder (ADHD), also including attention deficit disorder (without hyperactivity)	ICD-10: "F900" in the National Patient Registry and the Psychiatric Central Research Registry<br>ATC: "N06BA" in the Prescription Registry	Yes, if any relevant codes<br>No, otherwise
Depression	ICD-8: "29609" "29629" "29699" "29809" "30049" "30019" in the National Patient Registry or the Psychiatric Central Research Registry;<br>ICD-10: ICD-10: "F32" "F33" "F920" in the National Patient Registry or the Psychiatric Central Research Registry	Yes, if any relevant codes<br>No, otherwise
Anxiety disorder (including phobic disorders)	ICD-8: "30009" "30029" in the Patient Registry or The Psychiatric Central Research Registry;<br>ICD-10: "F40" "F41" "F931" "F932" "F9380" in the National Patient Registry or the Psychiatric Central Research Registry	Yes, if any relevant codes<br>No, otherwise
Epilepsy	ICD-8: "345" or<br>ICD-10: "G40" "G41" in the National Patient Registry	Yes, if any relevant codes<br>No, otherwise
Asthma	ICD-8: "493" in the Danish National Patient Registry<br>ICD-10: "J45" in the Patient Registry<br>ATC: "R03" in the Prescription Registry	Yes, if any diagnosis codes or two prescriptions for drugs against obstructive lung disease bronchodilators<br>No, otherwise
Rhinitis	ICD-8: "507";<br>ICD-10: "J30"<br>in the Patient Registry	Yes, if any relevant codes<br>No, otherwise
Preterm birth (<37 gestational week)	Based on the variables v_svlangde (in data table 't_lfoed'), Gestationsalder_uger (in data table 'Hjemmefoedsler_blanket'), or Gestationsalder_dage (in data table 'MFR') that encode gestational age (in weeks except the latter coded in days) in the Medical Birth Registry	Yes, if variable v_svlangde, Gestationsalder_uger, or Gestationsalder_dage/**7** is <37<br>No, otherwise

© 2021 Schmidt et al. *JAMA Dermatol.*

Low birth weight (<2500 grams)	Based on the variables V_vagt (table 't_lfoed'), vaegt_barn (table 'MFR'), or vaegt_barn (table 'Hjemmefoedsler_blanket') that encode birth weight in the Medical Birth Registry	Yes, if any of the variables has a value of <2500 g<br>No. otherwise
5-min Apgar score <7 or intrauterine/birth asphyxia	Based on the variables V_apgar5 (table 't_lfoed'), Apgarscore_efter5minutter (table 'MFR'), or Apgarscore_efter5minutter (table 'Hjemmefoedsler_blanket') that encode 5-min Apgar score in the Medical Birth Registry; ICD-8 code "776" or ICD-10 code "DP20" or "DP21" in the National Patient Registry	Yes, if variable V_apgar5 or Apgarscore_efter5minutter <5 or any of the listed ICD-8/10 codes.<br>No, otherwise
Chromosomal abnormalities (as defined by Eurocat)	ICD-8: "7593" "7594" "7595";<br>ICD-10: "Q9" in the Patient Registry	Yes, if any relevant codes.<br>No, otherwise
Parental income	PERINDKIALT_13 (during 1987-) and PERINDKIALT (1980-1986) in income data from Statistics Denmark	We categorised income in quartiles of income each year (to account for inflation) and then used the income of the parent with the highest income before baseline.
Parental educational level	As recorded in the Population Education Registry using the same codes for defining outcome (listed above).	As defined for outcome (lower secondary education, upper secondary education, higher education) using the educational level for the parent with the highest education.

Abbreviations: ICD=*International Classification of Diseases*; ATC=Anatomical Therapeutic Chemical classification system

Notes: All subcodes were included unless otherwise stated; all types of contacts (inpatient, outpatient, and emergency) and both primary and secondary diagnoses were considered. We used admission/prescription/record date for defining all

© 2021 Schmidt et al. *JAMA Dermatol*.

eMethods 2. Details of Sensitivity Analyses

We performed several sensitivity analyses to assess the robustness of our study results and assumptions. First, we adjusted additionally for potential mediators, including attention deficit (hyperactivity) disorder, depression and anxiety disorders, epilepsy, asthma, and rhinitis in regression models to examine if this further adjustment reduced the risk ratios. Second, we restricted the analyses to persons born on 30 June 1982 or earlier and determined study outcomes by age 35, as some persons may not have completed their education by age 30. Third, we excluded those who had adverse birth outcomes. Fourth, we repeated the main analysis for children with atopic dermatitis (and their comparators) who were also included in the sibling analyses (*i.e.*, children with atopic dermatitis who had at least one exposure-discordant full-sibling) to examine whether any difference between results from the main and sibling analyses could be explained by family structure (*e.g.*, exclusion of single-offspring families) or families without variability in the outcome among siblings. Fifth, because atopic dermatitis aggregates in families, we were concerned about a greater risk of misclassification of mild atopic dermatitis in sibling comparators. Therefore, we added the requirement that exposure-discordant siblings could have no prior prescription record (available since 1 January 1995) of a topical glucocorticoid/calcineurin inhibitor at baseline, as identified by Anatomical Therapeutic Chemical classification system codes D07, D11AH01, and D11AH02 in the Danish National Prescription Registry. Sixth, we repeated the sibling analysis after restricting it to siblings with a maximum age difference of 3 years, as some confounders (e.g., parental income and family structure) could change over time and thus differ between widely-spaced siblings. Finally, we also cross-tabulated parental income and educational level, respectively, at age 13 for siblings with vs. without atopic dermatitis, to examine the correlation of some family-related factors.

The results of the sensitivity analyses are provided in eTable 5 and eTable 6.

© 2021 Schmidt et al. *JAMA Dermatol*.

eFigure 1. Flowchart for Main Analysis

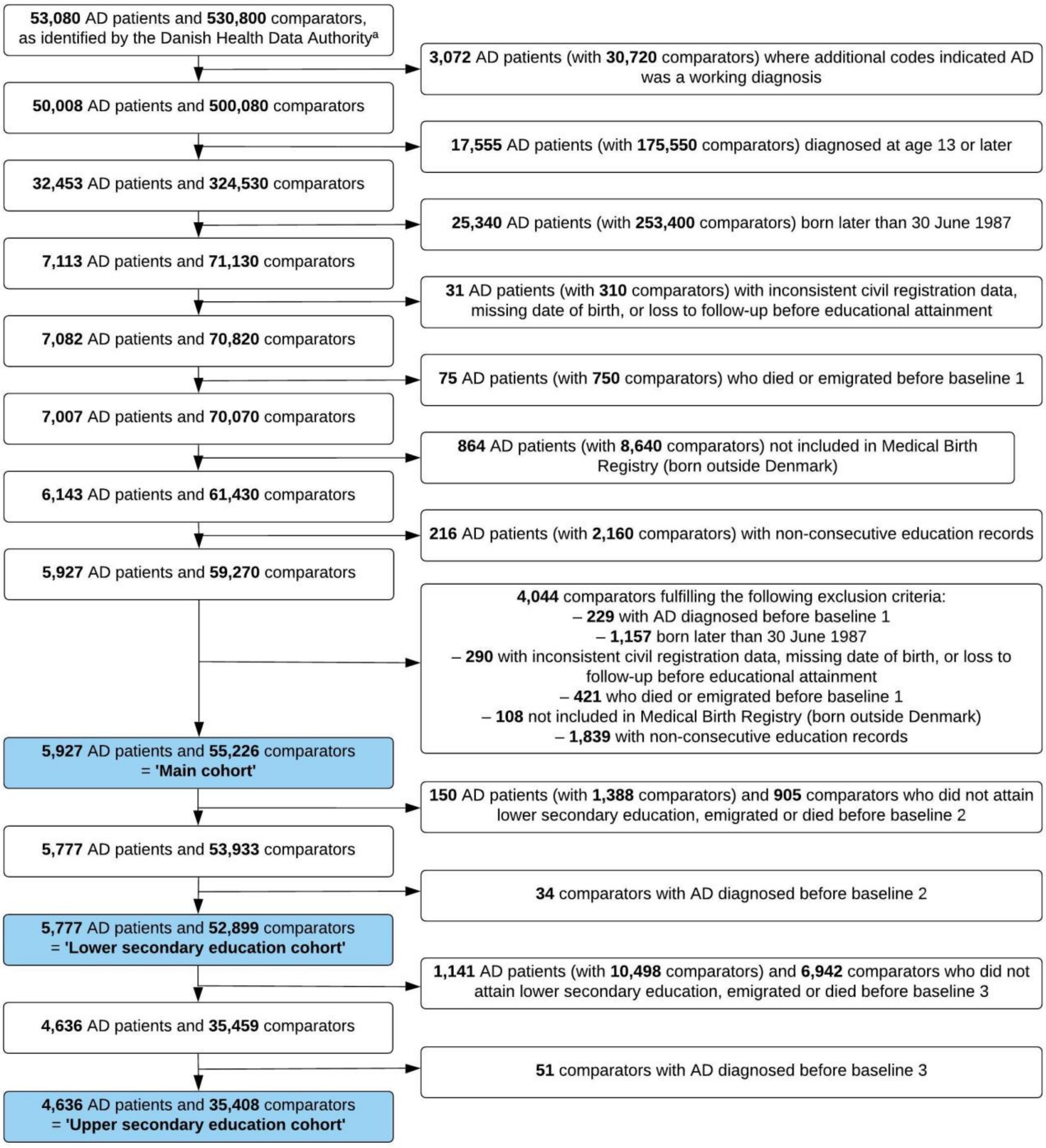

Abbreviations: AD=Atopic dermatitis

[a]The Danish Health Data Authority performed the initial sampling of cohorts. The AD cohort included persons with any primary or secondary inpatient, outpatient, or emergency room diagnosis of AD in the Danish National Patient Registry between 1 January 1977 and 10 February 2018. Only persons who were born in Denmark and living in Denmark on the AD diagnosis date (date of admission or first outpatient appointment) were included. The comparison cohort included up to 10 persons matched to each AD patient by sex and birth year. Comparators had to be born in Denmark, be alive and living in Denmark on the AD diagnosis date of their matched AD-exposed individual, and have no previous diagnosis of AD on this date.

© 2021 Schmidt et al. *JAMA Dermatol*.

eFigure 2. Flowchart for sibling analysis

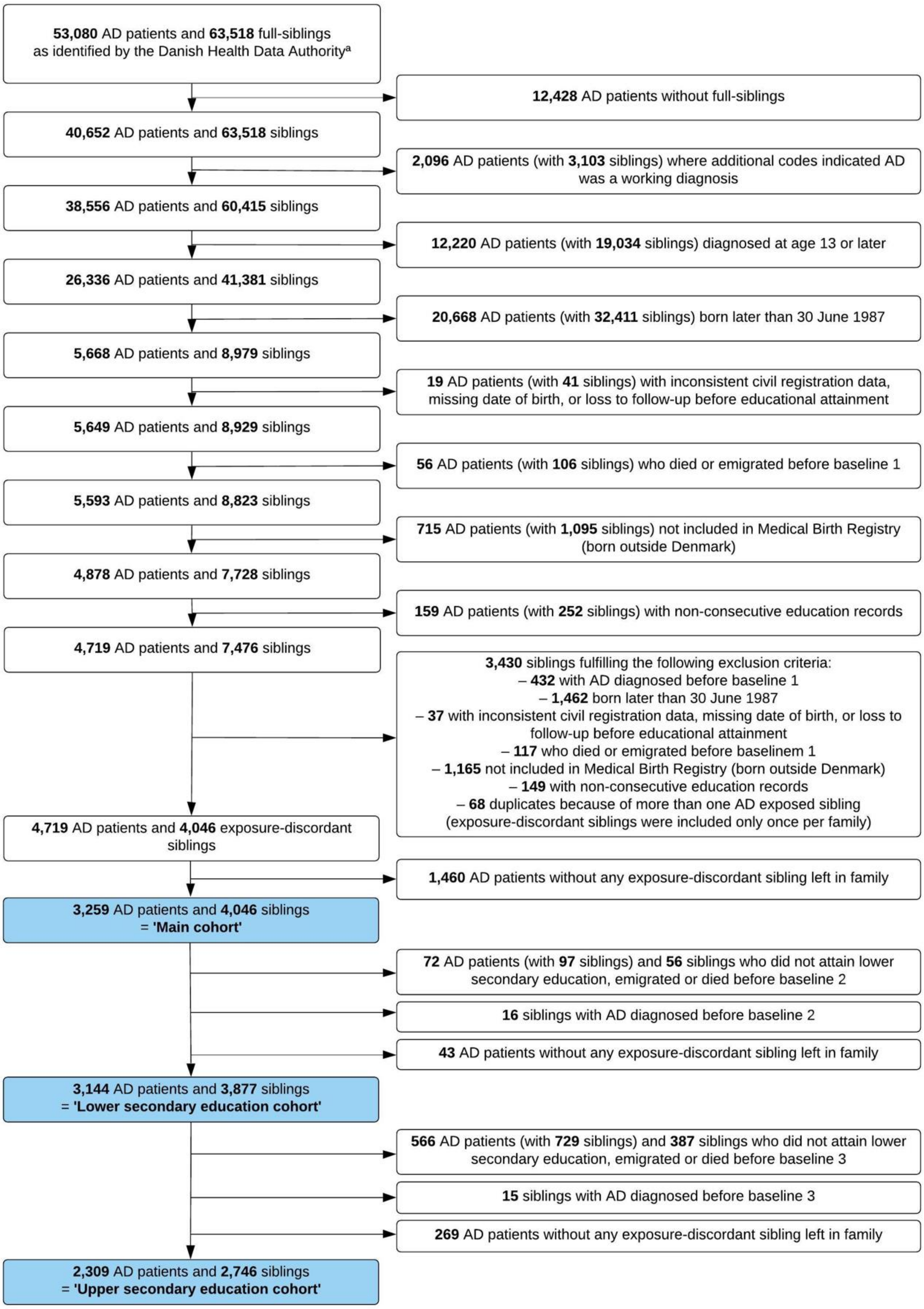

eFigure 2. Flowchart for Sibling Analysis

© 2021 Schmidt et al. *JAMA Dermatol.*

Abbreviations: AD=Atopic dermatitis
[a]The Danish Health Data Authority performed the initial sampling of cohorts. The AD cohort was defined as described in the footnote for eFigure 2 and full-siblings were defined as all persons with the same mother and father in the Civil Registration System.

© 2021 Schmidt et al. *JAMA Dermatol*.

eFigure 3. Probabilities and Risk Ratios (RRs) for not Attaining Specific Educational Levels

	AD, n (%)	Comparator, n (%)	Unadjusted RR (95% CI)[a]	Adjusted RR (95% CI)[b]
**Lower secondary education**				
Age at diagnosis				
– 0–4 years	55 (2.5)	60 (2.2)	1.25 (0.86, 1.81)	1.18 (0.80, 1.73)
– 5–12 years	17 (1.6)	12 (1.0)	1.77 (0.83, 3.79)	1.77 (0.83, 3.78)
Sex				
– Male	29 (2.8)	34 (2.8)	0.96 (0.58, 1.58)	0.96 (0.58, 1.58)
– Female	16 (2.1)	8 (0.9)	2.34 (0.99, 5.51)	2.34 (0.99, 5.51)
Parental income				
– Quartile 1	28 (5.1)	26 (3.7)	1.42 (0.83, 2.45)	1.29 (0.73, 2.27)
– Quartile 2	8 (1.7)	11 (2.1)	0.79 (0.32, 1.98)	0.92 (0.34, 2.46)
– Quartile 3	9 (1.9)	6 (1.1)	1.96 (0.69, 5.61)	1.78 (0.59, 5.37)
– Quartile 4	c	c	c	c
Parental education				
– Lower secondary	13 (4.1)	19 (4.7)	0.84 (0.41, 1.72)	0.81 (0.38, 1.71)
– Upper secondary	30 (2.1)	17 (1.0)	2.44 (1.33, 4.47)	2.37 (1.28, 4.39)
– Higher	8 (0.7)	14 (1.0)	0.76 (0.31, 1.84)	0.73 (0.30, 1.80)
**Upper secondary education**				
Age at diagnosis				
– 0–4 years	419 (19.7)	483 (18.2)	1.14 (1.00, 1.31)	1.09 (0.95, 1.25)
– 5–12 years	147 (14.4)	186 (15.2)	0.97 (0.78, 1.21)	0.95 (0.76, 1.19)
Sex				
– Male	204 (20.4)	241 (21.1)	1.02 (0.85, 1.24)	1.00 (0.83, 1.21)
– Female	118 (16.1)	116 (14.1)	1.16 (0.89, 1.50)	1.15 (0.89, 1.49)
Parental income				
– Quartile 1	188 (34.6)	212 (30.8)	1.17 (0.96, 1.43)	1.12 (0.91, 1.38)
– Quartile 2	88 (19.8)	81 (16.5)	1.19 (0.88, 1.61)	1.15 (0.85, 1.57)
– Quartile 3	59 (12.8)	66 (12.5)	1.04 (0.73, 1.49)	1.03 (0.71, 1.49)
– Quartile 4	58 (9.3)	52 (7.3)	1.27 (0.87, 1.85)	1.11 (0.74, 1.67)
Parental education				
– Lower secondary	102 (36.4)	130 (36.7)	1.04 (0.80, 1.36)	1.02 (0.78, 1.34)
– Upper secondary	282 (20.0)	316 (18.7)	1.10 (0.94, 1.30)	1.06 (0.89, 1.25)
– Higher	94 (8.0)	96 (6.7)	1.30 (0.97, 1.75)	1.13 (0.83, 1.54)
**Higher education**				
Age at diagnosis				
– 0–4 years	789 (51.7)	906 (49.6)	1.03 (0.94, 1.14)	0.97 (0.88, 1.07)
– 5–12 years	327 (41.7)	449 (48.9)	0.84 (0.72, 0.97)	0.88 (0.76, 1.02)
Sex				
– Male	397 (58.1)	426 (56.5)	1.02 (0.88, 1.17)	0.99 (0.86, 1.14)
– Female	224 (39.7)	248 (39.9)	0.99 (0.82, 1.19)	1.00 (0.83, 1.21)
Parental income				
– Quartile 1	260 (64.8)	319 (67.4)	0.97 (0.83, 1.15)	0.96 (0.81, 1.13)
– Quartile 2	188 (59.9)	195 (56.0)	1.04 (0.85, 1.28)	1.00 (0.81, 1.23)
– Quartile 3	124 (36.5)	158 (41.7)	0.86 (0.68, 1.09)	0.84 (0.66, 1.08)
– Quartile 4	133 (30.0)	159 (31.0)	0.90 (0.72, 1.14)	0.94 (0.74, 1.20)
Parental education				
– Lower secondary	89 (73.0)	112 (78.3)	0.92 (0.70, 1.22)	0.89 (0.67, 1.19)
– Upper secondary	592 (60.0)	712 (62.1)	0.96 (0.86, 1.07)	0.93 (0.83, 1.04)
– Higher	341 (32.1)	401 (31.6)	0.99 (0.86, 1.15)	0.98 (0.84, 1.14)

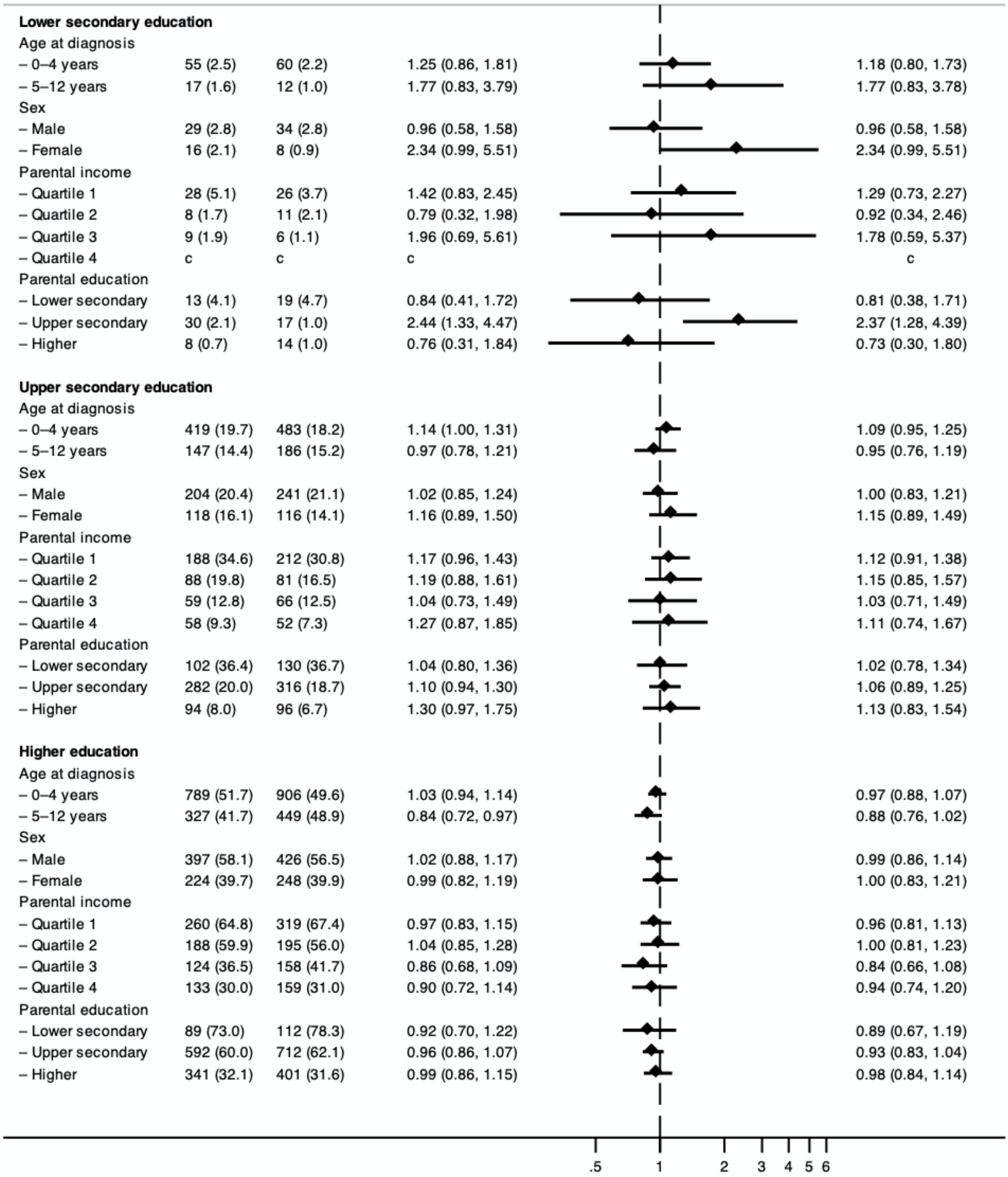

Probabilities and risk ratios (RRs) with 95% confidence intervals (CIs) for not attaining specific educational levels among children with atopic dermatitis (AD) compared with full-siblings without AD, stratified by age at AD diagnosis, sex, parental income and parental education.

[a]Accounting for family. Age at baseline is the same for all members of the main cohort.
[b]Additionally adjusted for age at baseline and sex.

© 2021 Schmidt et al. *JAMA Dermatol*.

eTable 1. Probability of Subtypes of Upper Secondary Education and Higher Education

Outcome	Main analysis				Sibling analysis			
	AD cohort		Comparison cohort		AD cohort		Sibling cohort	
	N	%	N	%	N	%	N	%
**Upper secondary education**								
Not achieving any type of upper secondary education	1141	19.8	8690	16.4	566	18.0	669	17.3
Achieving general upper secondary education	2948	51.0	28,038	53.0	1654	52.6	2099	54.1
Achieving vocational upper secondary education	1688	29.2	16171	30.6	924	29.4	1109	28.6
**Higher education**								
Not achieving any type of higher education	2406	51.9	18,785	53.1	1116	48.3	1355	49.3
Achieving short cycle higher education	384	8.3	3049	8.6	208	9.0	233	8.5
Achieving medium cycle higher education	875	18.9	6584	18.6	476	20.6	566	20.6
Achieving long cycle higher education	971	20.9	6990	19.7	509	22.0	592	21.6

Probability in children with atopic dermatitis, a matched general population comparison cohort, and a cohort of full-siblings.

© 2021 Schmidt et al. *JAMA Dermatol*.

eTable 2. Probability and Risk Ratios (RRs) for not Attaining Specific Educational Levels

A) Main analysis

Outcome	Analysis	No. of AD patients/ comparators	Not achieving educational level					
			AD		Comparators		Unadjusted RR (95% CI)[a]	Adjusted RR (95% CI)[b]
			No.	%	No.	%		
**Lower secondary education**	**Main result**	**5927/55,226**	**150**	**2.5**	**924**	**1.7**	**1.50 (1.26–1.78)**	**1.55 (1.27–1.88)**
	1. Adjusting additionally for ADHD, depression and anxiety disorder, epilepsy, asthma, and rhinitis	5927/55,226	150	2.5	924	1.7	NA	1.56 (1.28–1.89)
	2. Outcome assessed up to age 35 years (restricted to those born on or before June 30, 1982)	3431/31,830	79	2.3	499	1.6	1.47 (1.16–1.87)	1.47 (1.16–1.87)
	3. Excluding those with 5-min Apgar score<7, intrauterine/birth asphyxia, low birth weight, or chromosomal abnormalities	5310/44,923	125	2.4	728	1.6	1.44 (1.19–1.74)	1.44 (1.19–1.74)
	4. Restricted to the subset of AD patients (and their comparators) who were also included in the sibling analyses	3258/30,480	72	2.2	499	1.6	1.34 (1.05–1.72)	1.34 (1.05–1.72)
**Upper secondary education**	**Main result**	**5777/52,899**	**1141**	**19.8**	**8690**	**16.4**	**1.20 (1.13–1.28)**	**1.14 (1.06–1.22)**
	1. Adjusting additionally for ADHD, depression and anxiety disorder, epilepsy, asthma, and rhinitis	5777/52,899	1141	19.8	8690	16.4	NA	1.16 (1.08–1.24)
	2. Outcome assessed up to age 35 years (restricted to those born on or before June 30, 1982)	3352/30,599	624	18.6	4543	14.9	1.25 (1.15–1.36)	1.21 (1.11–1.31)
	3. Excluding those with 5-min Apgar score<7, intrauterine/birth asphyxia, low birth weight, or chromosomal abnormalities	5184/43,106	967	18.7	6845	15.9	1.18 (1.10–1.26)	1.14 (1.07–1.22)
	4. Restricted to the subset of AD patients (and their comparators) who were also included in the sibling analyses	3186/29,285	581	18.2	4768	16.3	1.12 (1.03–1.22)	1.09 (1.00–1.19)
**Higher education**	**Main result**	**4636/35,408**	**2406**	**51.9**	**18785**	**53.1**	**0.98 (0.94–1.02)**	**0.97 (0.92–1.01)**
	1. Adjusting additionally for ADHD, depression and anxiety disorder, epilepsy, asthma, and rhinitis	4636/35,408	2406	51.9	18785	53.1	NA	0.97 (0.92–1.01)
	2. Outcome assessed up to age 35 years (restricted to those born on or before June 30, 1982)	2728/21,134	1359	49.8	10851	51.3	0.97 (0.91–1.02)	0.96 (0.90–1.01)
	3. Excluding those with 5-min Apgar score<7, intrauterine/birth asphyxia, low birth weight, or chromosomal abnormalities	4216/29,365	2175	51.6	15547	52.9	0.97 (0.93–1.02)	0.95 (0.91–0.99)
	4. Restricted to the subset of AD patients (and their comparators) who were also included in the sibling analyses	2604/20,003	1326	50.9	10730	53.6	0.95 (0.89–1.00)	0.94 (0.89–1.00)

[a]Accounting for sex. Age at baseline is the same for all members of the main cohort. [b]Additionally adjusted for age at baseline. In sensitivity analysis 1, the adjusted estimate also includes ADHD, depression and anxiety disorder, epilepsy, asthma, and rhinitis.

B) Sibling analysis

Outcome	Analysis		Not achieving educational level

© 2021 Schmidt et al. *JAMA Dermatol*.

		No. of AD patients/ comparators	AD		Comparators		Unadjusted RR (95% CI)[a]	Adjusted RR (95% CI)[b]
			No.	%	No.	%		
**Lower secondary education**	**Main result**	**3259/4046**	**72**	**2.2**	**72**	**1.8**	**1.35 (0.96–1.88)**	**1.29 (0.92–1.82)**
	1. Adjusting additionally for ADHD, depression and anxiety disorder, epilepsy, asthma, and rhinitis	3259/4046	72	2.2	72	1.8	NA	1.55 (1.06–2.28)
	2. Outcome assessed up to age 35 years (restricted to those born on or before June 30, 1982)	1610/1868	39	2.4	33	1.8	1.66 (1.03–2.67)	1.58 (0.98–2.56)
	3. Excluding those with 5-min Apgar score <7, intrauterine/birth asphyxia, low birth weight, or chromosomal abnormalities	2794/3408	54	1.9	52	1.5	1.37 (0.93–2.02)	1.33 (0.89–1.97)
	5. Requiring that siblings had no prior prescription record of a topical steroid/calcineurin inhibitor at baseline	433/441	12	2.8	10	2.3	1.32 (0.57–3.07)	1.42 (0.58–3.49)
	6. Restricted to siblings with an age difference of 3 years or less	1603/1716	44	2.7	33	1.9	1.49 (0.95–2.35)	1.42 (0.88–2.30)
**Upper secondary education**	**Main result**	**3144/3877**	**566**	**18.0**	**669**	**17.3**	**1.10 (0.98–1.23)**	**1.05 (0.93–1.18)**
	1. Adjusting additionally for ADHD, depression and anxiety disorder, epilepsy, asthma, and rhinitis	3144/3877	566	18.0	669	17.3	NA	1.01 (0.89–1.16)
	2. Outcome assessed up to age 35 years (restricted to those born on or before June 30, 1982)	1557/1791	261	16.8	295	16.5	1.10 (0.93–1.30)	1.05 (0.88–1.25)
	3. Excluding those with 5-min Apgar score <7, intrauterine/birth asphyxia, low birth weight, or chromosomal abnormalities	2710/3285	459	16.9	544	16.6	1.07 (0.94–1.21)	1.02 (0.90–1.16)
	5. Requiring that siblings had no prior prescription record of a topical steroid/calcineurin inhibitor at baseline	342/348	63	18.4	58	16.7	1.11 (0.78–1.59)	1.08 (0.75–1.56)
	6. Restricted to siblings with an age difference of 3 years or less	1536/1639	312	20.3	331	20.2	1.05 (0.90–1.23)	1.01 (0.86–1.18)
**Higher education**	**Main result**	**2309/2746**	**1116**	**48.3**	**1355**	**49.3**	**0.97 (0.89–1.05)**	**0.94 (0.87–1.02)**
	1. Adjusting additionally for ADHD, depression and anxiety disorder, epilepsy, asthma, and rhinitis	2309/2746	1116	48.3	1355	49.3	NA	0.96 (0.87–1.06)
	2. Outcome assessed up to age 35 years (restricted to those born on or before June 30, 1982)	1164/1297	540	46.4	600	46.3	0.99 (0.88–1.12)	0.97 (0.86–1.10)
	3. Excluding those with 5-min Apgar score <7, intrauterine/birth asphyxia, low birth weight, or chromosomal abnormalities	2016/2366	955	47.4	1163	49.2	0.95 (0.87–1.03)	0.92 (0.85–1.01)
	5. Requiring that siblings had no prior prescription record of a topical steroid/calcineurin inhibitor at baseline	194/197	86	44.3	85	43.2	1.01 (0.75–1.37)	0.89 (0.64–1.24)
	6. Restricted to siblings with an age difference of 3 years or less	1058/1105	517	48.9	546	49.4	0.98 (0.87–1.11)	0.93 (0.82–1.06)

Probabilities among children with atopic dermatitis (AD) compared with a matched general population cohort (main analysis) and a cohort of full-siblings without AD (sibling analysis): sensitivity analyses,

.[a]Accounting for family. Age at baseline is the same for all members of the main cohort. [b]Additionally adjusted for age at baseline and sex. In sensitivity analysis 1, the adjusted estimate also includes ADHD, depression and anxiety disorder, epilepsy, asthma, and rhinitis.

© 2021 Schmidt et al. *JAMA Dermatol*.

eTable 3. Correlation Between Parental Income and Parental Educational Level Among Exposure-Discordant Siblings

A) 2x2 table of the level of parental income for AD patients and sibling(s) at baseline 1

	**AD**		**Sibling(s)**	
**Parental income level**	**N**	**%**	**N**	**%**
Quartile 1	775	23.8	1040	25.8
Quartile 2	822	25.3	1003	24.9
Quartile 3	824	25.3	997	24.7
Quartile 4	31	25.6	995	24.7

Notes: 18 had missing data

B) Difference in parental income category between AD patients and corresponding sibling(s) at baseline 1

**Difference in parental income category**	**Frequency**	**Percent**	**Cumulative frequency**	**Cumulative percent**
-3	44	1.1	44	1.1
-2	115	2.9	159	3.9
-1	620	15.4	779	19.3
0	2470	61.2	3249	80.5
1	611	15.1	3860	95.7
2	138	3.4	3998	99.1
3	37	0.9	4035	100.0

Notes: 11 had missing data on the difference.
The difference represents the difference in category of income, where income is coded as 1=income in quartile 1; 2= income in quartile 2; 3=income in quartile 3; and 4=income in quartile 4. Thus, a difference of -3 means that at baseline for the main cohort, the parental income category for the exposure-discordant sibling was 3 levels higher than parental income level of the AD-exposed sibling.

C) 2x2 table of level of parental educational level for AD patients and sibling(s) at baseline 1

	**AD**		**Sibling(s)**	
**Parental educational level**	**N**	**%**	**N**	**%**
Lower secondary education	325	10.9	417	11.4
Upper secondary education	1470	49.2	1786	48.8
Higher education	1196	40.0	1456	39.8

Notes: 655 had missing data

D) Differences between AD patient and sibling(s) and corresponding sibling(s) at baseline 1

**Difference in parental educational level**	**Frequency**	**Percent**	**Cumulative frequency**	**Cumulative percent**
-1	17	0.5	17	0.5
0	3600	98.7	3617	99.1
1	32	0.9	3649	100.0

Notes: 397 had missing data on the difference. The difference represents the difference in category of education, where educational level is coded as 1=lower secondary education; 2=upper secondary education; and 3=higher education. Thus, a difference of -1 means that at baseline for the main cohort, the parental educational level of the exposure-discordant sibling was one level higher than the parental educational level of the AD-exposed sibling.

© 2021 Schmidt et al. *JAMA Dermatol.*

eReferences

1. Schmidt M, Schmidt SAJ, Adelborg K, et al. The Danish health care system and epidemiological research: from health care contacts to database records. *Clin Epidemiol*. 2019;11:563-591. doi:10.2147/CLEP.S179083.

2. *The Danish Education System. the Ministry of Higher Education and Science, the Ministry for Children, Education and Gender Equality and the Ministry of Culture.*; 2016:1-24. ufm.dk/en/publications.

3. *State Educational Grant and Loan Scheme (SU)*. Ministry of Higher Education https://www.su.dk/english/state-educational-grant-and-loan-scheme-su/.

4. Schmidt M, Pedersen L, Sørensen HT. The Danish Civil Registration System as a tool in epidemiology. *Eur J Epidemiol*. 2014;29(8):541-549.

5. Schmidt M, Schmidt SAJ, Sandegaard JL, Ehrenstein V, Pedersen L, Sørensen HT. The Danish National Patient Registry: A review of content, data quality, and research potential. *Clin Epidemiol*. 2015;7:449-490.

6. Mors O, Perto GP, Mortensen PB. The Danish Psychiatric Central Research Register. *Scand J Public Health*. 2011;39(7 Suppl):54-57. doi:10.1177/1403494810395825.

7. Pottegård A, Schmidt SAJ, Wallach-Kildemoes H, Sørensen HT, Hallas J, Schmidt M. Data Resource Profile: The Danish National Prescription Registry. *Int J Epidemiol*. 2017;46(3):798–798f. doi:10.1093/ije/dyw213.

8. Bliddal M, Broe A, Pottegård A, Olsen J, Langhoff-Roos J. The Danish Medical Birth Register. *Eur J Epidemiol*. 2018;33(1):27-36. doi:10.1007/s10654-018-0356-1.

9. Jensen VM, Rasmussen AW. Danish Education Registers. *Scand J Public Health*. 2011;39(7 Suppl):91-94. doi:10.1177/1403494810394715.

10. Baadsgaard M, Quitzau J. Danish registers on personal income and transfer payments. *Scand J Public Health*. 2011;39(7 Suppl):103-105. doi:10.1177/1403494811405098.

11. Description of Education in Denmark. Ministry of Higher Education and Science. https://ufm.dk/en/education/higher-education/degrees-and-qualifications.

12. International Standard Classification of Education ISCED 2011. UNESCO Institute for Statistics. 2012:1-88.

13. Weidinger S, Beck LA, Bieber T, Kabashima K, Irvine AD. Atopic dermatitis. *Nat Rev Dis Primers*. 2018;4(1):1-20. doi:10.1038/s41572-018-0001-z.

14. Silverberg JI. Selected comorbidities of atopic dermatitis: Atopy, neuropsychiatric, and musculoskeletal disorders. *Clin Dermatol*. 2017;35(4):360-366. doi:10.1016/j.clindermatol.2017.03.008.

15. Miyazaki C, Koyama M, Ota E, et al. Allergic diseases in children with attention deficit hyperactivity disorder: a systematic review and meta-analysis. *BMC Psychiatry*. 2017;17(1):120. doi:10.1186/s12888-017-1281-7.

© 2021 Schmidt et al. *JAMA Dermatol.*

16. Patel KR, Immaneni S, Singam V, Rastogi S, Silverberg JI. Association between atopic dermatitis, depression, and suicidal ideation: A systematic review and meta-analysis. *J Am Acad Dermatol*. 2019;80(2):402-410. doi:10.1016/j.jaad.2018.08.063.

17. Young S, Hollingdale J, Absoud M, et al. Guidance for identification and treatment of individuals with attention deficit/hyperactivity disorder and autism spectrum disorder based upon expert consensus. *BMC Medicine*. 2020;18(1):146. doi:10.1186/s12916-020-01585-y.

18. Fleming M, Fitton CA, Steiner MFC, et al. Educational and health outcomes of children and adolescents receiving antidepressant medication: Scotland-wide retrospective record linkage cohort study of 766 237 schoolchildren. *Int J Epidemiol*. 2020;43:8. doi:10.1093/ije/dyaa002.

19. Chen M-H, Wu Y-H, Su T-P, et al. Risk of epilepsy among patients with atopic dermatitis: a nationwide longitudinal study. *Epilepsia*. 2014;55(8):1307-1312. doi:10.1111/epi.12667.

20. Silverberg JI, Joks R, Durkin HG. Allergic disease is associated with epilepsy in childhood: a US population-based study. *Allergy*. 2014;69(1):95-103. doi:10.1111/all.12319.

21. Wo SW, Ong LC, Low WY, Lai PSM. The impact of epilepsy on academic achievement in children with normal intelligence and without major comorbidities: A systematic review. *Epilepsy Res*. 2017;136:35-45. doi:10.1016/j.eplepsyres.2017.07.009.

22. Ruijsbroek A, Wijga AH, Gehring U, Kerkhof M, Droomers M. School Performance: A Matter of Health or Socio-Economic Background? Findings from the PIAMA Birth Cohort Study. Glymour MM, ed. *PLoS ONE*. 2015;10(8):e0134780. doi:10.1371/journal.pone.0134780.

23. Zhu T, Zhao J, Qu Y, Zhang L, Mu D. Association of very preterm birth with decreased risk of eczema: A systematic review and meta-analysis. *J Am Acad Dermatol*. 2018;78(6):1142-1148.e1148. doi:10.1016/j.jaad.2017.12.015.

24. Civelek E, Sahiner UM, Yüksel H, et al. Prevalence, burden, and risk factors of atopic eczema in schoolchildren aged 10-11 years: a national multicenter study. *J Investig Allergol Clin Immunol*. 2011;21(4):270-277.

25. Lundholm C, Ortqvist AK, Lichtenstein P, Cnattingius S, Almqvist C. Impaired fetal growth decreases the risk of childhood atopic eczema: a Swedish twin study. *Clin Exp Allergy*. 2010;40(7):1044-1053. doi:10.1111/j.1365-2222.2010.03519.x.

26. Moore MM, Rifas-Shiman SL, Rich-Edwards JW, et al. Perinatal predictors of atopic dermatitis occurring in the first six months of life. *Pediatrics*. 2004;113(3 Pt 1):468-474. doi:10.1542/peds.113.3.468.

27. Egeberg A, Andersen YMF, Gislason G, Skov L, Thyssen JP. Neonatal risk factors of atopic dermatitis in Denmark - Results from a nationwide register-based study. *Pediatr Allergy Immunol*. 2016;27(4):368-374. doi:10.1111/pai.12560.

© 2021 Schmidt et al. *JAMA Dermatol.*
